# Studies of the Functionalized α-Hydroxy-*p*-Quinone Imine Derivatives Stabilized by Intramolecular Hydrogen Bond

**DOI:** 10.3390/molecules29071613

**Published:** 2024-04-03

**Authors:** Anastasija Gaile, Sergey Belyakov, Ramona Dūrena, Ņikita Griščenko, Anzelms Zukuls, Nelli Batenko

**Affiliations:** 1 Institute of Chemistry and Chemical Technology, Faculty of Natural Sciences and Technology, Riga Technical University, P. Valdena Str. 3, LV-1048 Riga, Latvia; anastasija.gaile@rtu.lv; 2 Latvian Institute of Organic Chemistry, Aizkraukles Str. 21, LV-1006 Riga, Latvia; serg@osi.lv; 3 Institute of Materials and Surface Engineering, Faculty of Natural Sciences and Technology, Riga Technical University, P. Valdena Str. 3, LV-1048 Riga, Latvia; ramona.durena@rtu.lv (R.D.); nikita.griscenko@rtu.lv (Ņ.G.); anzelms.zukuls@rtu.lv (A.Z.)

**Keywords:** quinone, quinone imine, hydrogen bonding, X-ray crystallography, NMR titration, redox

## Abstract

In this work, reactions between 6,7-dichloropyrido[1,2-*a*]benzimidazole-8,9-diones with different benzohydrazides were studied. Nucleophilic substitution at C(6) was followed by isomerization and led to α-hydroxy-*p*-quinone imine derivatives. Synthesized compounds represent a combination of several structural motifs: a benzimidazole core fused with α-hydroxy-*p*-quinone imine, which contains a benzamide fragment. X-ray crystallography analysis revealed the formation of dimers linked through OH···O interactions and stabilization of the imine form by strong intramolecular NH···N hydrogen bonds. The protonation/deprotonation processes were investigated in a solution using UV–Vis spectroscopy and a ^1^H NMR titration experiment. Additionally, the electrochemical properties of 6,7-dichloropyrido[1,2-*a*]benzimidazole-8,9-dione and its α-hydroxy-*p*-quinone imine derivative as cathode materials were investigated in acidic and neutral environments using cyclic voltammetry measurements. Cathode material based on 6,7-dichloropyrido[1,2-*a*]benzimidazole-8,9-dione could act as a potentially effective active electrode in aqueous electrolyte batteries; however, further optimization is required.

## 1. Introduction

Quinones and quinone derivatives are well known due to the redox activity that is important in a wide range of biological processes, such as photosynthesis [1] and cellular respiration [2]. Quinones represent a class of biologically active compounds with both cytotoxic and cytoprotective effects [3]. Considerable attention to redox-active compounds in general and quinones in particular [4,5] can be explained by growing demands on energy storage devices for portable electronics and renewable energy-powered vehicles [6]. Quinones can potentially be used in different applications connected with energy storage due to their remarkable redox activity: as organic cathode materials for different kinds of rechargeable batteries [7], including redox flow batteries [8] and Zn-ion batteries [9], or as redox mediators in lithium–sulfur batteries [10]. Physical properties of quinones can be modulated by the introduction of heteroaromatics fused with quinone cores [8] and different substituents that affect solubility [11] or affect the form of quinone fragment [12] or can facilitate binding with metal cations [13]. Additionally, redox properties may be tuned to some extent by intra- and intermolecular hydrogen bonding [14].

Redox potentials, solubility, and stability in the case of small quinones can be affected by modification with electron-donating or -withdrawing functional groups or in combination with a side chain that can form hydrogen bonds. Additionally, substituted *o*-quinones, besides their “classical” form, can also exist in various forms [15], like quinone methides, quinone imines, and zwitterions. This ability to adopt different structures allows modulation of their properties for different applications. Despite progress in the design of quinone derivatives and their wide application as redox-active materials, limited information on molecular-level insights into the bulk properties (e.g., solubility, stability, redox activity, etc.) is available. The investigation of quinone structure at the molecular level, self-assembly in solid state, and behavior in solution will help tune the performance of quinone-functionalized materials.

An approach to modulate the redox properties of quinones is an introduction of nitrogen-containing redox-active groups (e.g., C≡N, C=N, and N=N) or incorporation of unsaturated carbon–nitrogen bonds and π-conjugated aromatic fragments [16].

This work aimed to gain an understanding of the molecular structure and behavior of unsymmetrical heterocyclic *o*-quinones and their derivatives bearing imine moiety. Also, electrochemical studies of selected compounds were conducted to assess their potential applications.

## 2. Results and Discussion

### 2.1. Synthesis and Structural Studies of Quinone Derivatives ***3a***–***g***

**Scheme 1:**
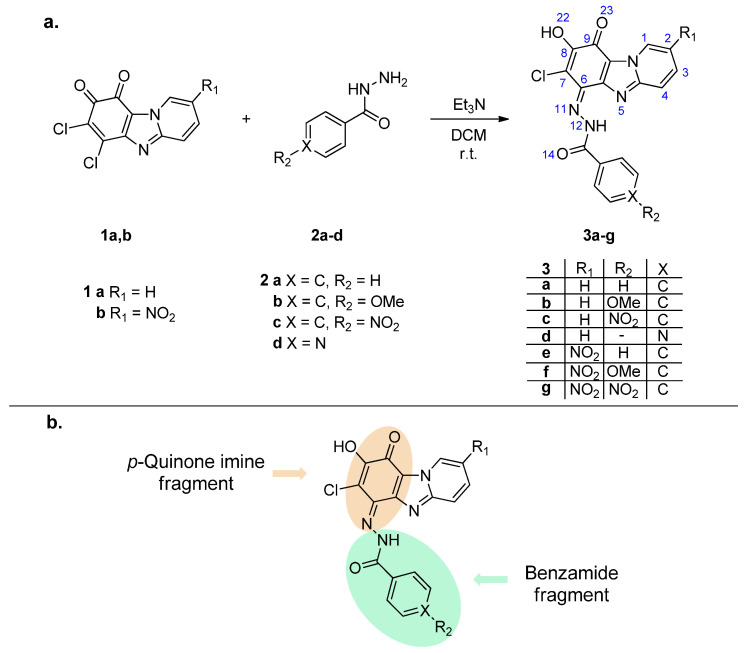
(**a**) Synthesis of compounds **3a**–**g,** atom numbering of heterocyclic *p*-quinone imine fragment of compounds **3a**–**g** is shown in blue; (**b**) *p*-Quinone imine and benzamide fragments of obtained compounds **3a**–**g**.

6,7-Dichloropyrido[1,2-*a*]benzimidazole-8,9-dione (**1a**) is a representative of unsymmetrical heterocyclic *o*-quinones that contains a combination of two structural motifs, an *o*-quinone fragment and imidazo[1,2-*a*]pyridine core, that possess C=N bonds (Scheme 1). It can be obtained in one-step synthesis from commercially available tetrachloro-1,4-benzoquinone and 2-aminopyridine [17]. During earlier studies [17,18,19], it was proved that quinone **1a** and some of its derivatives are electrochemically active compounds. Investigation of the reactivity of heterocyclic quinones **1** with *C*- and *N*-nucleophiles (primary amines) indicated that the attack of the nucleophile proceeds selectively at the C(6)-position of quinone. Interestingly, obtained compounds containing different acceptor groups at the C(6)-position can exist in an *o*-quinone form or as *p*-quinone methides, depending on the introduced substituent.

To expand the scope of redox-active heterocyclic *o*-quinone derivatives, the modification of quinone **1a** with different benzohydrazides was carried out, and structural studies in solid state and solution were conducted.

#### 2.1.1. Synthesis of Quinone Derivatives **3a**–**g**

Quinone derivatives **3a**–**g** (Scheme 1a, atoms are numbered according to ORTEP diagram, vide infra) were obtained by the nucleophilic substitution of a chlorine atom of quinone **1a**,**b** by benzohydrazides **2a**–**d**. Isolated compounds **3a**–**g** have a red-orange color in the solid state. Interestingly, in the case of aminoderivatives of quinone **1** (a merocyanine on the base of the *o*-quinone form), deep-blue colored crystals were obtained [17,19]. In general, derivatives containing aroyl hydrazine fragments were expected as a result of such substitution [15,20], and a few tautomeric structures can be supposed for the products [21,22]. For the compounds obtained (**3a**–**g**), the structure determination of the quinone/substituent fragments (Scheme 1b) can explain the observed difference in color of crystals **3a**–**g** in comparison to aminosubstituted derivatives of quinone **1a**.

#### 2.1.2. Single-Crystal X-ray Analysis of Quinone Derivative **3a**

The use of routine identification procedures (such as ^1^H-NMR and FTIR) to establish the molecular structure of compounds **3a**–**g** left some room for doubt. To clarify the situation, crystals of compound **3a** were grown from dichloromethane (DCM) solution, and their molecular structure was established using single-crystal X-ray crystallography. Crystal data and refinement details for the studied crystal are presented in Table 1.

**Figure 1:**
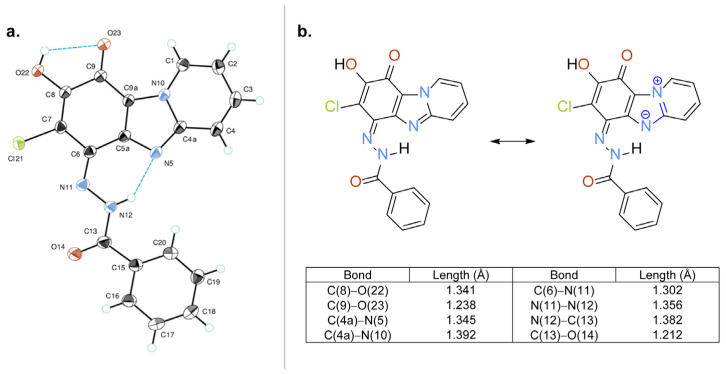
(**a**) ORTEP diagram of compound **3a** showing thermal ellipsoids at the 50% probability level; (**b**) two mesomeric structures of compound **3a** based on the bond distances in the crystal structure.

Figure 1a shows a perspective view of molecule **3a** with thermal ellipsoids and the atom-numbering scheme. The *p*-quinone imine form was confirmed by the inspection of the bond length: bonds between C(9)=O(23) and C(6)=N(11) have double-bond character, and O(22)-C(8) is a single bond (typical bond distances: *d (*Å) (C-O) = 1.34, (C=O) = 1.21, (C-N) = 1.38, (C=N) = 1.28 [23]). Also, an analysis of bond lengths shows that the structure of compound **3a** can be represented as a superposition of mesomeric forms. The main forms are shown in Figure 1b; at that, the non-ionized form has the highest specific weight.

**Figure 2:**
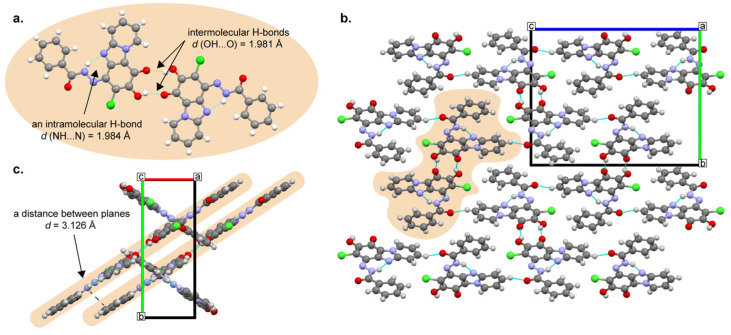
(**a**) A dimer (highlighted) formed in the crystal structure of compound **3a**. (**b**) Crystal packing of compound **3a** along the *a* axis. (**c**) Crystal packing of compound **3a** along the *c* axis. Color map: C, grey; N, blue; O, red; Cl, green; H, white.

The molecules of compound **3a** are characterized by a flattened conformation; only the phenyl group is slightly out of the plane of the heterocyclic system (the angle between planes is 5.19°). In the structure of compound **3a,** intramolecular hydrogen bonds NH···N and OH···O were found (Figure 2). The hydroxy group of compound **3a** forms bifurcated hydrogen bonds where O(22)-H···O(23) is an intramolecular bond (Figure 1a) and O(22)-H···O’(23) is an intermolecular one (Figure 2a). By means of these intermolecular H-bonds, the centrosymmetric $R_{2}^{2}(10)$ molecular dimers are formed in the crystal structure. Additional intermolecular interactions were found: stacking interaction between the planes of the molecules and a short intermolecular contact between heterocycle (C(3)-H) and the amide group of the substituent (*d* C(3)···O(14) = 3.112 Å, *d* C(3)-H···O(14) = 2.344 Å) (Figure 2b,c).

Hirshfeld surfaces and energy framework calculations (Figures S14 and S15) were obtained in a whole-of-molecule approach using the B3LYP/6-31G(d,p) energy model implemented in CrystalExplorer 21.5 software [24]. The Hirshfeld surface (shown in Figure S14, mapped with d_norm_) investigation is a useful approach to establishing close contacts in a crystal. Areas of close contact are highlighted with different colors (red, white, and blue spots) to show intermolecular contacts with distances less than, equal to, and larger than van der Waal radii. As seen from the Hirshfeld surface analysis of compound **3a**, bright red areas of the same size indicate strong interactions, specifically hydrogen bonds OH···O, between neighboring molecules. Fainter red or white areas suggest relatively weak interactions involving hydrogen atoms from heterocyclic and phenyl rings and chlorine atoms. Examples include C-H bonds interacting with oxygen (C(3)-H···O(14)), nitrogen (C(3)-H···N(11)), and chlorine (C(4)-H··· Cl and C(phenyl)-H⋯Cl).

Energy frameworks provide an opportunity to explore the cooperative effects of intermolecular interactions in the crystal, admitting the electrostatic, dispersion, and total energy between pairs of molecules [25]. In the case of the crystal of compound **3a** (Figure S15), a strong stabilizing interlayer electrostatic interaction was found between molecules involved in the formation of hydrogen-bonded (O–H⋯O) dimers. On the other hand, dispersion energy was more dominant for the intercolumn stacking motif. Overall, the energy framework analysis of the crystal revealed two distinct patterns of electrostatic and dispersion energies, with each contributing similarly.

#### 2.1.3. ^1^H NMR Spectroscopy Analysis of Quinone Derivatives **3a**–**g**

To determine the structure of obtained products in solution compounds **3a**–**g,** ^1^H NMR spectroscopy data were analyzed, and a set of two broad signals corresponding to NH and OH protons was observed (Figures S1–S7). In the DMSO-*d*_6_ solution, signals appeared at 14.36–14.90 ppm and can be assigned to the NH proton, while signals of the OH group were observed at 10.89–11.41 ppm. Additionally, the ^1^H NMR spectrum of compound **3a** was also recorded in CDCl_3_ solution (a solvent in which hydrogen-bonding interactions are expected to be weaker [26]) (Figure S8), where the NH proton was found at 14.68 ppm (versus 14.71 ppm in DMSO-*d*_6_ solution). It can be concluded that a strong intramolecular bond between the NH group proton of the substituent (benzamide group at imine bond) and the nitrogen of the heterocycle (N(12)-H···N(5)) can be found in solution as well as in a solid state (vide supra).

It is known [27] that in the case of α-hydroxyquinone derivatives, an intramolecular hydrogen bond was observed, and an OH proton signal appears at 7.30 ppm in the CDCl_3_ solution. For compound **3a**, a distinguishable shift was observed for the OH proton signal in the CDCl_3_ solution (7.20 ppm) in comparison to the DMSO-*d*_6_ solution (10.99 ppm), which can indicate the formation of the additional intermolecular interactions between the OH group and a solvent (DMSO-*d*_6_) with hydrogen bond acceptor abilities [28].

Moreover, the introduction of various substituents in the phenyl ring (benzamide fragment) showed the linear correlation (Figure S9) between the type of substituent group (represented by the Hammett constant [29] (Table S1)) and OH signal shift in the sets of compounds **3a**–**c** (R_1_ = H) and **3e**–**g** (R_1_ = NO_2_). The shift of the OH signal correlates well (R^2^ = 0.99) with the increasing electron-withdrawing character of the substituent group for both sets of compounds (Figure S9). The presence of an electron-withdrawing group (EWG) at the heterocyclic fragment (R_1_ = NO_2_) led to a downfield shift in the OH signal compared to molecules without any substituent (R_1_ = H) on that ring (Δδ_OH_ ≈ 0.3 ppm).

The most downfield signal of the NH proton (14.90 ppm in DMSO-*d*_6_ solution) in the ^1^H NMR spectra was observed for compound **3c** with a NO_2_ group at the phenyl ring (R_2_ at benzamide fragment). The most upfield signal of the NH proton (14.36 ppm) was found for compound **3f** with a NO_2_ group at the heterocyclic fragment (R_1_) and electron-donating group at the phenyl ring (R_2_ = OMe). Well-defined linear correlations between the Hammett substituent constant and the NH signal are presented in Figure S9. The variation in the electronic character of the substituent in the *para*-position of the phenyl ring showed the same trend for compounds **3a**–**c** (R^2^ = 0.96) and **3e**–**g** (R^2^ = 0.99), leading to the downfield shift of the NH signal going from an electron-donating to electron-withdrawing group. Interestingly, the presence of EWG (R₁ = NO₂) at the heterocyclic ring caused the NH signal upfield shift (Δδ_NH_ ≈ 0.3 ppm) compared to a molecule where the same position has a hydrogen atom (R₁ = H). In general, the more downfield shifted the NH proton signal, the stronger the intramolecular H-bond [30]. Thus, EWG at the phenyl ring (R_2_ = NO_2_) increases the acidity of the NH proton and increases the intramolecular H-bond strength, but EWG at the heterocyclic fragment (R_1_ = NO_2_) influences the electron density at N(5), affecting intramolecular H-bond in turn.

In the case of compounds **3a**–**g**, the moiety at the C(6) position can be described as a structural analog of aroyl hydrazone (a different approach [22] to the naming of quinone imine derivatives was observed). A well-known characteristic of compounds containing the carbon–nitrogen double bond is the ability to undergo *E*/*Z* isomerization in the solution activated by light and/or chemical inputs [31,32].

Compounds **3a** and **3b** were chosen for the investigation of the isomerization process. In general, in the case of *E*/*Z* isomerization, an additional set of signals [33,34] is expected to appear. No signals of the second form (isomerization products) were observed in the ^1^H NMR spectra of compound **3a** either in DMSO-*d*_6_ or in CDCl_3_ solution. Also, in the case of compound **3b**, configurational switching was not induced by the addition of excess trifluoracetic acid (TFA) and following irradiation by UV light (365 nm; irradiation by a high-pressure mercury lamp at room temperature) judging from the ^1^H NMR spectra of compound **3b** in DMSO-*d*_6_ solution (Figure S10). The formation of a strong intramolecular hydrogen bond N(12)-H···N(5) can explain the existence of a single configuration of substituted imine that agrees with the stabilization of only one form in the presence of an intramolecular hydrogen bond. Additional stabilization of the molecule may be explained by excitation energy dissipation caused by zwitterionic structure.

It is known [35] that for redox properties tests (fabrication of electrodes), a mixture of an organic compound, a conductive additive, and a binder is often prepared using *N*-methyl-2-pyrrolidone (NMP) [36] as a solvent (strongly basic solvent) [37]. This fact prompted us to investigate the influence of the base on the structure of the products **3a**–**g**.

**Figure 3:**
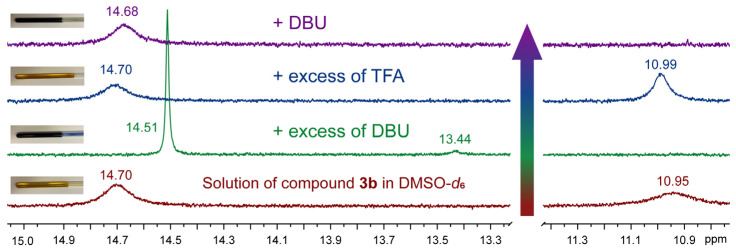
An expansion of ^1^H NMR spectra of compound **3b** in DMSO-*d*_6_ solution upon sequential addition of base (DBU) and acid (TFA).

During preliminary solubility tests of compounds **3a**–**g,** the color change (from yellow to green or blue) was observed in NMP solution or in the presence of a base. For a better understanding of the effect, a few ^1^H NMR experiments were carried out. Upon addition of an excess of the base (1,8-diazabicyclo(5.4.0)undec-7-ene, DBU), the ^1^H NMR spectrum of compound **3b** in DMSO-*d*_6_ solution showed some changes (Figure 3): the signal of OH proton completely disappeared; the sharpened signal of the NH proton (the sharp line can indicate a dynamically stable state) shifted upfield. Additionally, a new minor proton signal at 13.44 ppm was observed. Simultaneously, the yellow-colored solution of compound **3b** became dark blue. It should be noted that the same deprotonation behavior was observed for compound **3a** in CDCl_3_ solution (Figure S11). After the excess TFA was added, the solution became yellow again, a minor signal at 13.44 ppm disappeared, and the signal of the OH proton was restored. It can be concluded that deprotonation provides the formation of an anionic polymethine dye structure [38] (blue), and protonation restores quinone imine form (yellow); consequently, the equilibrium between the two forms is reversible.

**Figure 4:**
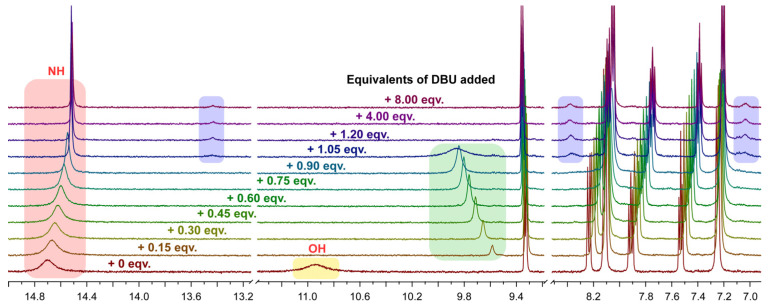
^1^H NMR titration of compound **3b** in DMSO-*d*_6_ solution with DBU (0–8 equivalents).

To gain more information about the deprotonation process of compound **3b**, a ^1^H NMR titration experiment was carried out. As shown in Figure 4, in the case of compound **3b** deprotonation upon sequential addition of the base (DBU), the ^1^H NMR spectra reveal several features. The broad signal of the OH proton vanished upon the addition of only 0.15 equivalents of the base, which can be explained by the dynamical process as well; color changes were immediate (Figure 4, highlighted in yellow). The signal of the NH proton (benzamide fragment) sharpens and undergoes an upfield shift from 14.70 to 14.51 ppm (Figure 4, highlighted in red).

A new signal appeared at 9.58 ppm (+0.15 eqv. of DBU) (Figure 4, highlighted in green), which can be explained by the formation of a hydrogen-bonded complex of protonated DBU (^1^H NMR spectrum of DBU and TFA mixture in DMSO-*d*_6_ solution was recorded; the NH^+^ signal appears at 9.69 ppm (Figure S12)) with the deprotonated compound **3b**. Upon further addition of the base, this signal was broadened and shifted downfield (9.86 ppm) due to the interaction of protonated DBU (DBUH^+^) with the anionic compound **3b** through the N–H bond [39].

Upon addition of more than 1.05 eqv. of the base, a second minor form of the compound appears (Figure 4, highlighted in blue); the ratio between major and minor forms is 0.95:0.05, taking into consideration the signals of all protons. Moreover, the addition of an excess amount of the base (4 and 8 eqv.) did not result in the change of the ^1^H NMR spectra of compound **3b** (additional processes as a function of the time and/or temperature in the solution should not be excluded as ^1^H NMR titration experiment was carried out within an hour after addition of DBU to the compound **3b** at room temperature (T = 294 K)).

Also, the presence of two different species (one major and one minor) was detected from the changes in the ^1^H NMR spectra of compounds **3a** and **3c** upon deprotonation with DBU in DMSO-*d*_6_ solution (after mixed with more than 1 equivalent of DBU). For compound **3a,** the ^1^H NMR spectrum was also recorded in the presence of NaOH. As a result, the acquired spectrum was identical to the one with an excess of DBU (Figure S13).

Unfortunately, the low solubility of compounds **3a**–**g** limited the possibility of obtaining qualitative ^13^C NMR spectra.

#### 2.1.4. Electronic Absorption

**Figure 5:**
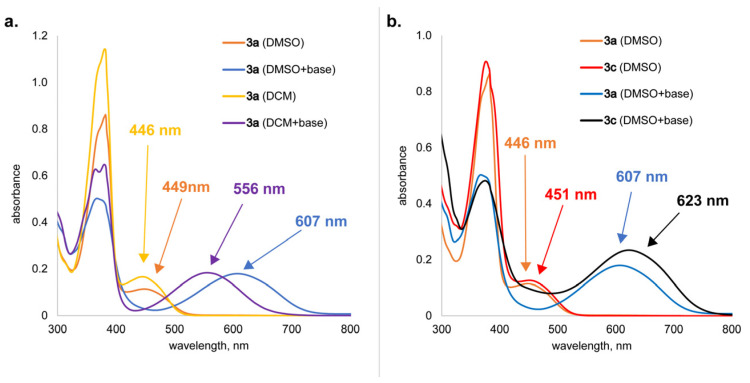
(**a**) UV–Vis absorption spectra of neutral (in DMSO or DCM solution) and deprotonated (upon addition base to the DMSO or DCM solution) forms of compound **3a**. (**b**) UV–Vis absorption spectra neutral (in DMSO solution) and deprotonated (upon addition of a base to the DMSO solution) forms of compounds **3a** and **3c**.

The UV–Vis absorption spectra of compound **3a** were investigated in solution using solvents of various polarities (DCM and DMSO). Two absorption maxima were found at 381 nm and at 446–449 nm in the absorption spectra of compound **3a** in both solutions (Figure 5a), which can be attributed to the neutral form of the compound. Upon addition of the base (DBU) to the DCM solution of **3a**, the solution instantaneously turned violet, and the absorption revealed a broad band centered at 556 nm. When a base was added to the DMSO solution of compound **3a**, the bathochromic shift was observed with an absorption maxima at 607 nm accompanied by a blue coloration.

The effect of substituents (R_1_ and R_2_) on the longwave absorption maximum was examined when DBU was added to the initial solution of compounds **3a**–**c** (variable R_2_ while R_1_ = H) and **3e**–**g** (variable R_2_ while R_1_ = NO_2_) in CHCl_3_ (Figure S16, Table S2). It was noticed that the longwave absorption band of deprotonated species of derivatives **3e**–**g** was red-shifted (λ = 569–578 nm) in comparison to compounds **3a**–**c** (λ = 556–563 nm). This observation can be explained by the influence of the electron-withdrawing substituent at C(2) on the electron distribution in the heterocyclic fragment. Moreover, a hyperchromic effect was caused by the introduction of the nitro group in the benzamide fragment (compound **3c** in Figure 5b; compounds **3c** and **3g** in Figure S16).

### 2.2. Electrochemistry/Redox Chemistry Studies of Quinone Derivatives ***1a*** and ***3a***

Quinone imines contain structural fragments with potentially high redox activity. It was shown by Almeida, R. et al. [40] that *p*-quinone imines are known to undergo a redox cycle through aminophenols [41]. Compound **1a** was previously proved to be electrochemically active [42] when dissolved in the MeCN solution. However, it has not been tested as a cathode material before. To analyze the redox properties of quinone imine **3a** in comparison to initial *o*-quinone **1a**, open-circuit potential (OCP) and cyclic voltammetry (CV) measurements in a solid state were carried out. Preliminary solubility tests showed limited solubility of compounds **1a** and **3a** in aqueous media; compound **1a** was insoluble in water (whole pH range), and at the same time, compound **3a** was insoluble in neutral and acidic environments.

#### 2.2.1. Open-Circuit Potential Measurements

To analyze the electrochemical properties of the compounds, CV measurements and OCP measurements before and after CV were performed (Figure S17). Cathode materials **CM-1a** and **CM-3a** were prepared by combining compounds **1a** and **3a** with Vulcan XC72 CB, respectively (the detailed sample preparation is described in the Experimental section). The OCP of freshly assembled half-cells for cathode materials **CM-1a** and **CM-3a** in the acidic (0.5 M H_2_SO_4_) electrolyte was 0.44 V and 0.37 V vs. Ag/AgCl; however, in a neutral (0.5 M K_2_SO_4_) electrolyte, the potentials of both were 0.28 V vs. Ag/AgCl. After the CV measurements, the OCP of sample half-cells stabilized. For both samples in the acidic electrolyte, they were 0.41 V vs. Ag/AgCl, and in the neutral electrolyte, they were 0.37 V vs. Ag/AgCl. The OCP for both samples is approximately the same, and with decreasing pH, there is a visible shift to higher potential values going from neutral to the acidic electrolyte.

#### 2.2.2. Cyclic Voltammetry Measurements

**Figure 6:**
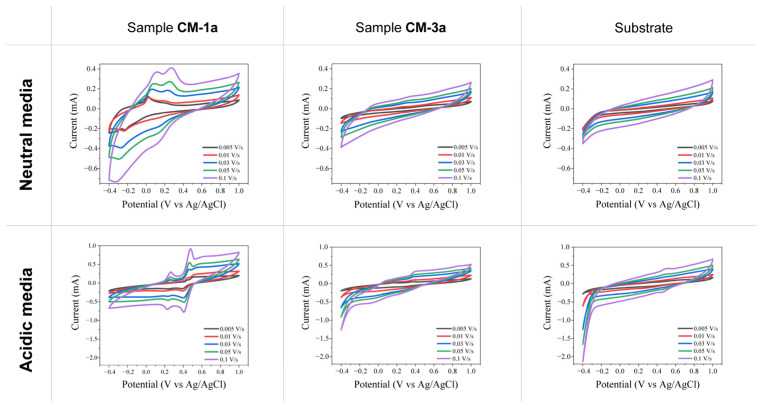
CV results at varying scanning speeds for samples **CM-1a**, **CM-3a**, and substrate in neutral (0.5 M K_2_SO_4_) electrolyte and in acidic (0.5 M H_2_SO_4_) electrolyte.

CV results for samples **CM-1a**, **CM-3a**, and a sample without active material (substrate) in neutral and acidic electrolytes at varying scanning speeds are shown in Figure 6. For the substrate in neutral electrolyte, no visible redox processes were observed. In the acidic electrolyte, a hydrogen evolution reaction can be observed around the potential of −0.3 V vs. Ag/AgCl and one insignificant redox process at faster scan rates around 0.3 V vs. Ag/AgCl. However, when scanning samples with active materials, this substrate process cannot be observed and, therefore, has no electrochemical significance other than providing electrical conductivity.

For sample **CM-3a** in a neutral electrolyte (Figure 6), no significant redox processes can be observed. Also, in an acidic electrolyte for sample **CM-3a** (Figure 6), there are no significant processes; however, upon closer inspection (Figure S18), two reversible oxidation (at 0.40 V and 0.07 V vs. Ag/AgCl) and reduction processes (at 0.23 V and −0.06 V vs. Ag/AgCl) can be seen.

Sample **CM-1a** has two redox maxima in the scanned potential window from −0.4 V to 1.0 V vs. Ag/AgCl electrode in both neutral and acidic electrolytes (Figure 6). In a neutral electrolyte (Figure S19), oxidation peaks are at 0.27 V and 0.10 V vs. Ag/AgCl; however, reduction peaks are at 0.14 V and −0.32 V vs. Ag/AgCl. In addition, the oxidation peaks are found at 0.48 V and 0.26 V vs. Ag/AgCl and reduction peaks at 0.40 V and 0.23 V vs. Ag/AgCl in an acidic electrolyte (Figure S20). This indicates a shift in reaction potential to more positive values by increasing H^+^ ion concentration and thus lowering the pH level of the electrolyte. Both redox processes for sample **CM-1a** correspond to the *o*-quinone fragment in the molecule. However, by comparing the electrochemical performance of both samples **CM-1a** and **CM-3a**, it can be concluded that by converting *o*-quinone **1a** to α-hydroxy-*p*-quinone imine **3a** accompanied by the additional stabilization by intra- and intermolecular hydrogen bonds, the electrochemical reactivity of the cathode material has been greatly suppressed.

All CV measurement result developments in time can be seen in Figure S21. At the start, samples were cycled at the potential window of −0.4 V to 1.0 V vs. Ag/AgCl reference electrode. Observations indicate that all samples go through the surface activation phase, where the sample-specific capacity increases with each subsequent cycle. During the measurements at different scanning speeds, the stabilization of the system is observed. However, sample **CM-1a** in neutral electrolyte goes through an irreversible oxidation process at 0.11 V vs. Ag/AgCl and a reduction process at −0.32 V vs. Ag/AgCl. This irreversible redox process can be observed during all scan speeds. At an increased potential window (from −1.0 V to 1.5 V), another irreversible process at a scan speed of 0.1 V/s for sample **CM-1a** in a neutral electrolyte can be observed during the oxidation at −0.31 V and reduction at −0.56 V vs. Ag/AgCl. A slight capacity decrease due to the possible dissolution of active materials can be observed for sample **CM-3a** in a neutral electrolyte and sample **CM-1a** in an acidic electrolyte.

#### 2.2.3. Raman Measurements

**Figure 7:**
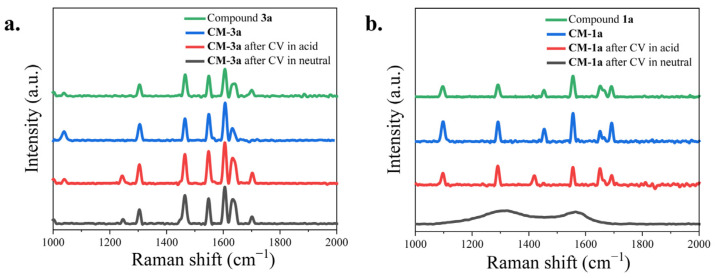
(**a**) Raman spectra of pure compound **3a** (green line), prepared cathode **CM-3a** before (blue line) and after cycling in acidic (red line) and neutral (gray line) electrolytes. (**b**) Raman spectra of pure compound **1a** (green line), prepared cathode **CM-1a** before (blue line) and after cycling in acidic (red line) and neutral (gray line) electrolytes.

Raman measurements were performed on pure compounds **1a** and **3a**, prepared cathodes (**CM-1a** and **CM-3a**), and after cycling them in acidic and neutral electrolytes (Figure 7). For sample **CM-3a**, the spectra for prepared and cycled cathodes remain as for pure compound **3a**, where an amide band can be seen at 1600–1630 cm^−1,^ as well as bands for aromatic/heteroaromatic rings at 1550 and 1470 cm^−1^ [43]. This indicates that compound **3a** was preserved in the cathode-forming process and did not go through any chemical changes. Also, after CV measurements, the active material is unchanged and present in the samples. For sample **CM-1a**, the spectra for the prepared and cycled cathode in an acidic electrolyte remain as for pure compound **1a** (bands for aromatic/heteroaromatic rings at 1570 and 1450 cm^−1^ and band for carbonyl groups at 1650–1690 cm^−1^) [43]. However, for cathode **CM-1a** cycled in neutral electrolyte, only C and D bands of carbon [44,45] can be seen. Since redox processes are visible for this sample in CV measurements (Figure 6), the active material could have dissolved from the electrode in the electrolyte and gone through the electrochemical reactions from the electrolyte.

#### 2.2.4. Scanning Electron Microscopy Measurements

Scanning electron microscopy examinations of compounds **1a** and **3a** (Figure S22) were performed to assess the morphology of the products. Compound **1a** consists of needle-like particles with sizes ranging from 1 µm to 10 µm in diameter and 3 µm to 30 µm in length. Compound **3a** has smaller particles with an overall size of 1 µm in diameter and 5–20 µm in length.

**Figure 8:**
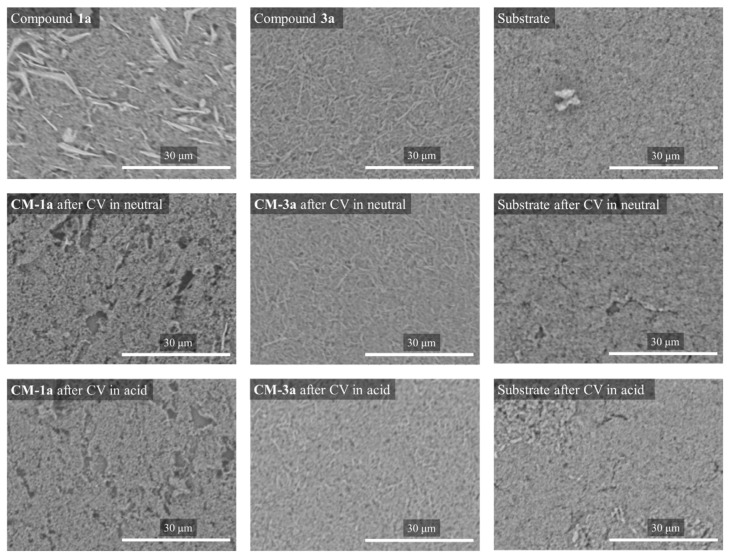
Scanning electron microscopy images of samples **CM-1a**, **CM-3a**, and substrate (coating without active material) before and after CV measurements in neutral and acidic electrolytes (magnification of ×2500).

Also, the prepared cathode surfaces with and without active materials before and after cyclic voltammetry are shown in Figure 8. The resemblance of the structures of compounds **1a** and **3a** (as shown in Figure S22) can be seen in the images of cathode disks (samples **CM-1a** and **CM-3a** in Figure 8) before CV measurements. For sample **CM-3a**, these structures can also be seen in images after CV in neutral and acidic electrolytes with some partial dissolution in an acidic electrolyte, as fewer structures can be seen. The formation of non-covalent interactions between compound **3a** and substrate can probably explain the greater stability of **CM-3a** in comparison to **CM-1a**. However, for sample **CM-1a** cycled in neutral and acidic electrolytes, only a few original structures can be seen. This correlates with findings from Raman spectroscopy (Figure 7b) that the active material **1a** dissolves in a neutral electrolyte and goes through electrochemical reactions from it.

## 3. Materials and Methods

### 3.1. Materials and Instrumentation

Polyvinylidene fluoride (PVDF) (MW ~530,000) and Dimethylformamide (DMF) were purchased from Merck; Vulcan XC72 Carbon Black (CB) was used, and 0.05 mm thick conductive graphite paper (RERAS, purchased from China and used as electrode substrate) was used to prepare cathode materials.

Melting points were measured on a Kruess KSP 11 Melting Point Analyzer. ^1^H NMR spectra were recorded on a Brucker Avance spectrometer at 300 or 500, respectively, in DMSO-*d_6_* or CDCl_3_ solutions. Chemical shifts were expressed in parts per million (δ, ppm) relative to solvent signal (DMSO-*d_6_*: 2.50 ppm CDCl_3_: 7.26 ppm for ^1^H NMR) [46]. Compounds **3a**–**g** are too insoluble to record a qualitative ^13^C NMR spectrum. Elemental CHN analysis was carried out on a Euro Vector EA 3000 analyzer. FTIR spectra were recorded on a Perkin-Elmer Spectrum 100 FTIR spectrometer. The UV–Vis absorption spectra were acquired with a Perkin-Elmer 35 UV/Vis spectrometer using 1 cm length quartz cuvettes with a concentration of compound *c* = 2.5 × 10^−5^ M. Low-resolution mass spectra were acquired on a Waters EMD 1000MS mass detector (ESI + mode, voltage 30 V) with an Xterra MS C18 5 μm 2.1 × 100 mm column and gradient eluent mode using 0.1% HCOOH in deionized water and MeCN or MeOH.

### 3.2. X-ray Crystallography Analysis

For compound **3a**, diffraction data were collected at a low temperature (T = 150.0(1) K) on Rigaku, XtaLAB Synergy, Dualflex, HyPix diffractometer using copper monochromated Cu-Kα radiation (λ = 1.54184 Å). The crystal structure was solved with the help of the ShelXT structure solution program [47] using the Intrinsic Phasing solution method. The model was refined with version of the program olex2.refine using Levenberg–Marquardt minimization [48]. All nonhydrogen atoms were refined in anisotropical approximation. For further details, see crystallographic data for compound **3a** deposited at the Cambridge Crystallographic Data Centre as Supplementary Publications Numbers CCDC 2238663 (for compound **3a**). These data can be obtained free of charge via https://www.ccdc.cam.ac.uk/structures/ (accessed on 1 April 2024) or from the CCDC, 12 Union Road, Cambridge CB2 1EZ, U.K.; Fax: +44 1223 336033; e-mail: deposit@ccdc.cam.ac.uk). For crystal packing visualization, the program *Mercury* [49] was used.

### 3.3. Cathode Material Preparation

Quinone derivatives **1a** or **3a** were combined with Vulcan XC72 CB at a mass ratio of 5:4. The resulting powder was dried overnight at 80 °C. Then, a binder solution of PVDF:DMF (mass ratio 1:9) was added so the quinone to PVDF mass ratio would be 5:1. Stirring and ultra-sonication were used to create the ink slurry. Extra DMF was added to the slurry to form homogeneous ink (the total weight ratio of DMF to quinone was approximately 12:1). A manual doctor blade coater (with a 25 µm gap size) was employed to apply the coating onto carbon paper that was pre-dried at 120 °C for an hour. Coated cathode substrates were then dried in air to evaporate DMF. For further material characterization, cathode disks were cut out using a hollow punch.

### 3.4. Cyclic Voltammetry

To analyze the electrochemical properties of the different sample compounds, cyclic voltammetry (CV) measurements were performed using a 3-electrode measuring cell “TSC Surface” (from rhd instruments). For different measurements, the prepared thin-layer electrodes on carbon paper (with or without compound **1a** or **3a**) were used as working electrodes placed in 1 mL of electrolyte with the platinum counter electrode and Ag/AgCl (3 M KCl) reference electrode. Two different pH electrolyte solutions were used for measurements: neutral 0.5 M K_2_SO_4_ and acidic 0.5 M H_2_SO_4_ solutions. The CV measurements were performed from −0.4 to +1.0 V with the following program: (1) open-circuit measurement (OCP) of freshly assembled half-cell before the CV measurements; (2) ten cycles with scan speed of 0.075 V/s to stabilize the half-cell; (3) 5 cycles of 5 scans with scan rates ranging from 0.005 V/s to 0.1 V/s; and (4) OCP measurement after CV.

### 3.5. Raman Spectroscopy

Raman measurements were performed using a Renishaw In-ViaV727 spectrometer in a backscattering geometry at room temperature at 100× magnification. For phonon excitation, a red laser (He-Ne, λ = 633 nm, grating—1200 mm^−1^, 125 µW) was used, and the sample exposure time was 10 s.

### 3.6. Scanning Electron Microscopy

A Hitachi TM3000 Tabletop scanning electron microscope (SEM) with an acceleration voltage of 5 kV was used to obtain surface information of the obtained electrode and sample materials. To characterize the obtained samples, different magnifications were used.

### 3.7. Synthesis of Quinone Derivatives ***1a***,***b*** and ***3a***–***g***

6,7-Dichloropyrido[1,2-a]benzimidazole-8,9-dione (**1a**) and 6,7-dichloro-2-nitropyrido[1,2-*a*]benzimidazole-8,9-dione (**1b**) were prepared according to the previously reported procedure [17,42].

General method for synthesis of compounds **3a**–**g**. To a stirring solution of 6,7-dichloropyrido[1,2-*a*]benzimidazole-8,9-dione (**1a**, 1 eq) or 6,7-dichloro-2-nitropyrido[1,2-*a*]benzimidazole-8,9-dione (**1b**, 1 eq) in dichloromethane (DCM) at room temperature, a solution of benzhydrazide derivative (**2a**–**d**, 2 eq) in DCM or DMF was added. Triethylamine (1 eq) was added to the reaction mixture, which was then stirred at room temperature for 8 h. After completion of the reaction, the reaction mixture was filtered through a filter paper, and the solvent was distilled in vacuo to a residual volume of 20 mL. The resulting orange-colored precipitate was collected, recrystallized from DCM/*n*-hexane, washed with hot ethanol (20 mL), and dried at room temperature.

**Compound 3a.** Prepared using 6,7-dichloropyrido[1,2-*a*]benzimidazole-8,9-dione (150 mg, 0.56 mmol, 1 eq), benzohydrazide (153 mg, 1.12 mmol, 2 eq), and triethylamine (*d* = 0.73 g/mL, v = 78 μL, 0.56 mmol, 1 eq). **Yield:** 59%, orange powder. **M.P.**: 258–260 °C. **MS:** C_18_H_11_ClN_4_O_3_ requires [M + H]^+^ 367.1; found [M + H]^+^ 367.2. **^1^H NMR (500 MHz, DMSO-*d_6_*):** 14.71 (br.s., 1H, exchange with D_2_O, NH), 10.98 (br.s., 1H, exchange with D_2_O, OH), 9.29 (d, *J* = 6.6 Hz, 1H, H-1), 8.16 (d, *J* = 9.0 Hz, 1H, H-4), 8.11 (d, *J* = 7.4, 2H, CH_Ph_), 7.89 (t, *J* = 8.0, 1H, H-3), 7.73 (m, 3H, CH_Ph_), 7.51 (d, *J* = 6.8 Hz, 1H, H-2). **FTIR (KBr, cm^−1^):** 3331, 3094, 3033, 1708, 1628, 1604, 1548, 1437, 1351, 1247. **Anal. Calcd.** for C_18_H_11_ClN_4_O_3_: C, 58.95; H, 3.02; N, 15.28; found C, 58.82; H, 3.02; N, 15.31.

**Compound 3b.** Prepared using 6,7-dichloropyrido[1,2-*a*]benzimidazole-8,9-dione (150 mg, 0.56 mmol, 1 eq), 4-methoxybenzohydrazide (187 mg, 1.12 mmol, 2 eq), and triethylamine (*d* = 0.73 g/mL, v = 78 μL, 0.56 mmol, 1 eq). **Yield:** 36%, orange solid. **M.P.**: >300 °C. **MS:** C_19_H_13_ClN_4_O_4_ requires [M + H]^+^ 397.1; found [M + H]^+^ 397.2. **^1^H NMR (500 MHz, DMSO-*d_6_*):** 14.70 (br.s., 1H, exchange with D_2_O, NH), 10.95 (br.s., 1H, exchange with D_2_O, OH), 9.33 (d, *J* = 6.7 Hz, 1H, H-1), 8.24 (d, *J* = 9.0 Hz, 1H, H-4), 8.11 (d, *J* = 8.6 Hz, 2H, CH_Ph_), 7.91 (m, 1H, H-3), 7.53 (t, *J* = 6.8 Hz, 1H, H-2), 7.25 (d, *J* = 8.5 Hz, 2H, CH_Ph_), 3.91 (s, 3H, -OCH_3_). **FTIR (KBr, cm^−1^):** 3468, 3301, 3083, 3024, 2975, 2832, 1690, 1630, 1609, 1582, 1552, 1504, 1351, 1325, 1259. **Anal. Calcd.** for C_19_H_13_ClN_4_O_4_: C, 57.51; H, 3.30; N, 14.12; found C, 57.58; H, 3.35; N, 13.82.

**Compound 3c.** Prepared using 6,7-dichloropyrido[1,2-*a*]benzimidazole-8,9-dione (150 mg, 0.56 mmol, 1 eq), 4-nitrobenzohydrazide (204 mg, 1.12 mmol, 2 eq), and triethylamine (*d* = 0.73 g/mL, v = 78 μL, 0.56 mmol, 1 eq). **Yield:** 52%, orange solid. **M.P.**: 275–278 °C. **MS:** C_18_H_10_ClN_5_O_5_ requires [M + H]^+^ 412.1; found [M + H]^+^ 412.2. **^1^H NMR (300 MHz, DMSO-*d_6_*):** 14.95 (br.s., 1H, exchange with D_2_O, NH), 11.07 (br.s., 1H, exchange with D_2_O, OH), 9.29 (d, *J* = 6.5, 1H, H-1), 8.53 (m, 2H, CH_Ph_), 8.32 (d, *J* = 8.3, 3H, H-4 un CH_Ph_), 7.91 (m, 1H, H-3), 7.53 (m, 1H, H-2). **FTIR (KBr, cm^−1^):** 3618, 3306, 3113, 3089, 3016, 1703, 1626, 1605, 1573, 1556, 1519, 1346, 1275. **Anal. Calcd.** for C_18_H_10_ClN_5_O_5_: C, 52.51; H, 2.45; N, 17.01; found C, 52.20; H, 2.58; N, 16.73.

**Compound 3d.** Prepared using 6,7-dichloropyrido[1,2-*a*]benzimidazole-8,9-dione (150 mg, 0.56 mmol, 1 eq), isonicotinohydrazide (154 mg, 1.12 mmol, 2 eq), and triethylamine (*d* = 0.73 g/mL, v = 78 μL, 0.56 mmol, 1 eq). **Yield:** 50%, orange crystals. **M.P.**: >250 °C (decomp.). **MS:** C_17_H_10_ClN_5_O_3_ requires [M + H]^+^ 368.1; found [M + H]^+^ 368.2. **^1^H NMR (300 MHz, DMSO-*d_6_*):** 14.52 (br.s., 1H, exchange with D_2_O, NH), 10.90 (br.s., 1H, exchange with D_2_O, NH), 9.31 (d, *J* = 6.7, 1H, H-1), 8.91 (d, *J* = 3.8, 2H, CH_Py_), 8.18 (d, *J* = 8.9, 1H, H-4), 7.97 (d, *J* = 4.6, 2H, CH_Py_), 7.89 (t, *J* = 8.0, 1H, H-3), 7.52 (t, *J* = 6.7, 1H, H-2). **FTIR (KBr, cm^−1^):** 3420, 3055, 1709, 1647, 1568, 1555, 1331, 1280. **Anal. Calcd.** for C_17_H_10_ClN_5_O_3_ + 0.5H_2_O: C, 54.20; H, 2.94; N, 18.59; found C, 54.29; H, 2.88; N, 18.30.

**Compound 3e.** Prepared using 6,7-dichloro-2-nitropyrido[1,2-a]benzimidazole-8,9-dione (150 mg, 0.48 mmol, 1 eq), benzohydrazide (131 mg, 0.96 mmol, 2 eq), and triethylamine (*d* = 0.73 g/mL, v = 67 μL, 0.48 mmol, 1 eq). **Yield:** 61%, yellow solid. **M.P.**: >250 °C (decomp.). **MS:** C_18_H_10_ClN_5_O_5_ requires [M + H]^+^ 412.1; found [M + H]^+^ 412.4. **^1^H NMR (300 MHz, DMSO-*d_6_*):** 14.42 (br.s., 1H, exchange with D_2_O, NH), 11.30 (br.s., 1H, exchange with D_2_O, OH), 10.06 (d, *J* = 2.3 Hz, 1H, H-1), 8.56 (dd, *J* = 9.8, 2.1 Hz 1H, H-3), 8.40 (d, *J* = 9.7 Hz, 1H, H-4), 8.12 (m, 2H, CH_Ph_), 7.72 (d, *J* = 7.7, 3H, CH_Ph_). **FTIR (KBr, cm^−1^):** 3397, 3091, 3033, 1690, 1642, 1552, 1525, 1351, 1311, 1268. **Anal. Calcd.** for C_18_H_10_ClN_5_O_5_: C, 52.51; H, 2.45; N, 17.01; found C, 52.21; H, 2.67; N, 16.76.

**Compound 3f.** Prepared using 6,7-dichloro-2-nitropyrido[1,2-a]benzimidazole-8,9-dione (150 mg, 0.48 mmol, 1 eq), 4-methoxybenzohydrazide (160 mg, 0.96 mmol, 2 eq), and triethylamine (*d* = 0.73 g/mL, v = 67 μL, 0.48 mmol, 1 eq). **Yield**: 42%, yellow crystals. **M.P.**: >250 °C (decomp.). **MS:** C_19_H_12_ClN_5_O_6_ requires [M + H]^+^ 442.1; found [M + H]^+^ 442.2. **^1^H NMR (300 MHz, DMSO-*d_6_*):** 14.36 (br.s., 1H, exchange with D_2_O, NH), 11.23 (br.s., 1H, exchange with D_2_O, OH), 10.05 (s, 1H, H-1), 8.56 (d, *J* = 9.7 Hz, 1H, H-3), 8.42 (d, *J* = 10.8 Hz, 1H, H-4), 8.10 (d, *J* = 7.1 Hz, 2H, CH_Ph_), 7.24 (d, *J* = 8.3 Hz, 2H, CH_Ph_), 3.90 (s, 3H, OCH_3_). **FTIR (KBr, cm^−1^):** 3399, 3085, 3028, 1687, 1640, 1605, 1555, 1523, 1350, 1310, 1266. **Anal. Calcd.** for C_19_H_12_ClN_5_O_6_: C, 51.66; H, 2.74; N, 15.85; found C, 51.35; H, 2.68; N, 15.61.

**Compound 3g.** Prepared using 6,7-dichloro-2-nitropyrido[1,2-a]benzimidazole-8,9-dione (150 mg, 0.48 mmol, 1 eq), 4-nitrobenzohydrazide (174 mg, 0.96 mmol, 2 eq), and triethylamine (*d* = 0.73 g/mL, v = 67 μL, 0.48 mmol, 1 eq). **Yield**: 36%, orange solid. **M.P.**: >250 °C (decomp.). **MS:** C_18_H_9_ClN_6_O_7_ requires [M + H]^+^ 457.0; found [M + H]^+^ 457.1. **^1^H NMR (300 MHz, DMSO-*d_6_*):** 14.61 (br.s., 1H, exchange with D_2_O, NH), 11.41 (br.s., 1H, exchange with D_2_O, OH), 10.03 (s, 1H, H-1), 8.58 (d, *J* = 10.8 Hz, 2H, H-4 and H-3), 8.51 (m, 2H, CH_Ph_), 8.32 (d, *J* = 8.3 Hz, 2H, CH_Ph_). **FTIR (KBr, cm^−1^):** 3340, 3114, 3081, 1707, 1626, 1602, 1547, 1518, 1344, 1306, 1266. **Anal. Calcd.** for C_18_H_9_ClN_6_O_7_: C, 47.33; H, 1.99; N, 18.40; found C, 47.24; H, 2.02; N, 18.14.

## 4. Conclusions

To summarize, a set of heterocyclic α-hydroxy-*p*-quinone imine derivatives was obtained via one-step nucleophilic substitution of 6,7-dichloropyrido[1,2-*a*]benzimidazole-8,9-diones **1a**,**b** with different benzohydrazides **2a**–**d** followed by isomerization. The α-Hydroxy-*p*-quinone imine form of the synthesized products was proved by X-ray crystallography analysis of compound **3a**. The formation of a strong intramolecular hydrogen bond N(12)-H···N(5) in a solid state and in solution can explain the stabilization of only one configuration of the C=N bond of the substituted imine. The ^1^H NMR acid-base titration experiment showed that deprotonation/protonation processes are reversible. Deprotonation led to the electronic delocalization in the molecule, which is accompanied by distinct changes in the UV–Vis spectra.

Sample **CM-1a** has a distinct maximum of two redox reactions. In an acidic environment, both peaks are stable, while in a neutral environment, only one of them is stable and remains unchanged after several CV cycles. Moreover, for the stable redox reactions, the potential difference is only up to 0.2 V. This indicates that the sample **CM-1a** could act as an effective active electrode in aqueous electrolyte batteries. However, the dissolution of the sample in the electrolyte was observed, so the potential application as cathode material for aqueous batteries would require compound **1a** to be attached to the polymer backbone.

Attempts to modulate redox properties by incorporation of additional unsaturated carbon–nitrogen bonds to the heterocyclic quinone **1a** structure led to changes in the redox-active fragment and the formation of *p*-quinone imine **3a**. As a result, the electrochemical behavior is changed, as it is no longer possible to observe pronounced redox peaks in CV measurements. Structural changes of the quinone fragment (probably induced by the formation of intramolecular H-bond) decreased the redox activity of the derivative despite the introduction of an additional C=N bond; at the same time, the introduced substituent enhanced the stability of the cathode material, which was confirmed by Raman spectroscopy measurements. Therefore, further structure optimizations for the elaboration of effective cathode material should include the analysis of the possible tautomerization, the formation of intramolecular H-bonds, the effect of substituents and interaction with basic solvents versus redox properties, and the stability of the compound.

## Figures and Tables

**Table 1:** Crystal data and structure refinement details for compound **3a**.

Crystal Parameter	Compound 3a
Empirical formula	C_18_H_11_ClN_4_O_3_
Calculated density (g/cm^3^)	1.556
Formula weight	366.766
Color	Red
Size/mm^3^	0.18 × 0.03 × 0.01
Temperature/K	150.0 (1)
Crystal system	monoclinic
Space group	*P*2_1_/*n*
*a*/Å	5.65994 (5)
*b*/Å	14.90948 (19)
*c*/Å	18.5815 (2)
α/°	90
β/°	93.1950 (9)
γ/°	90
*V*/Å^3^	1565.60 (3)
Wavelength/Å	1.54184
Radiation type	Cu K_α_
Absorption coefficient (mm^−1^)	2.419
θmin/°	3.8
2θmax/°	155.0
Measured reflections	17875
Number of independent reflections	3322
Reflections with I ≥ 2σ(*I*)	3089
*R_int_*	0.0327
Number of refined parameters	243
Restraints	0
Largest peak	0.3476
Deepest hole	−0.3143
Goodness of fit	1.0362
*wR_2_*(all data)	0.0962
*wR* *_2_*	0.0945
*R_1_*(all data)	0.0367
*R* *_1_*	0.0346
CCDC deposition number	2238663

## Data Availability

Data are contained within the article or Supplementary Materials.

## Supplementary Materials

The following supporting information can be downloaded at: https://www.mdpi.com/article/10.3390/molecules29071613/s1. Figures S1–S8: ^1^H NMR spectra (for compounds **3a**–**g** in DMSO-*d*_6_ solution and for compound **3a** in CDCl_3_ solution). Figure S9 and Table S1: Correlations between the Hammett constants (σ_p_) of substituent (R_2_) and the chemical shifts of NH or OH signals of compounds **3a**–**c** and **3e**–**g**. Figure S10: Additional ^1^H NMR spectra for compound **3b** upon acid addition and irradiation. Figures S11 and S13: ^1^H NMR spectra for compounds **3a**–**c** upon base addition. Figure S12: ^1^H NMR spectrum of DBU and TFA mixture. Figures S14 and S15: Hirshfeld surfaces and energy frameworks calculated with CrystalExplorer software. Figure S16 and Table S2: UV–Vis spectroscopy data of compounds **3a**–**c**, **3e**–**g**. Figures S17–S22: CV curves of samples **CM-1a** and **CM-3a** in neutral and acidic electrolytes at various scan speeds (PDF).
