# Supplementary material for: Studies of the Functionalized α-Hydroxy-p-Quinone Imine Derivatives Stabilized by Intramolecular Hydrogen Bond
Source: Molecules. 2024 Apr 3;29(7):1613. doi: 10.3390/molecules29071613 (PMC11013408; doi:10.3390/molecules29071613)
Supplement: Supplementary file 1 [file molecules-29-01613-s001.zip › molecules-2927989-supplementary.pdf]

Supporting Information

Studies of the Functionalized *α*-Hydroxy-*p*-Quinone Imine Derivatives Stabilized by Intramolecular Hydrogen Bond

Anastasija Gaile,[1] Sergey Belyakov,[2] Ramona Dūrena,[3] Ņikita Griščenko,[3] Anzelms Zukuls,[3]

Nelli Batenko[1,*]

[1]Institute of Chemistry and Chemical Technology, Faculty of Natural Sciences and Technology, Riga Technical University, P. Valdena Str. 3, Riga, LV-1048, Latvia

[2]Latvian Institute of Organic Chemistry, Aizkraukles Str. 21, Riga LV-1006, Latvia

[3]Institute of Materials and Surface Engineering, Faculty of Natural Sciences and Technology, Riga Technical University, P. Valdena Str. 3, Riga, LV-1048, Latvia

* **Corresponding Author**

E-mail address: nelli.batenko@rtu.lv (Nelli Batenko)

Table of content:

1. NMR spectroscopy data S2
2. Crystal Explorer: Hirshfeld Surfaces and Energy frameworks S9
3. UV-Vis spectroscopy data S10
4. Redox properties S11

1. NMR spectroscopy data

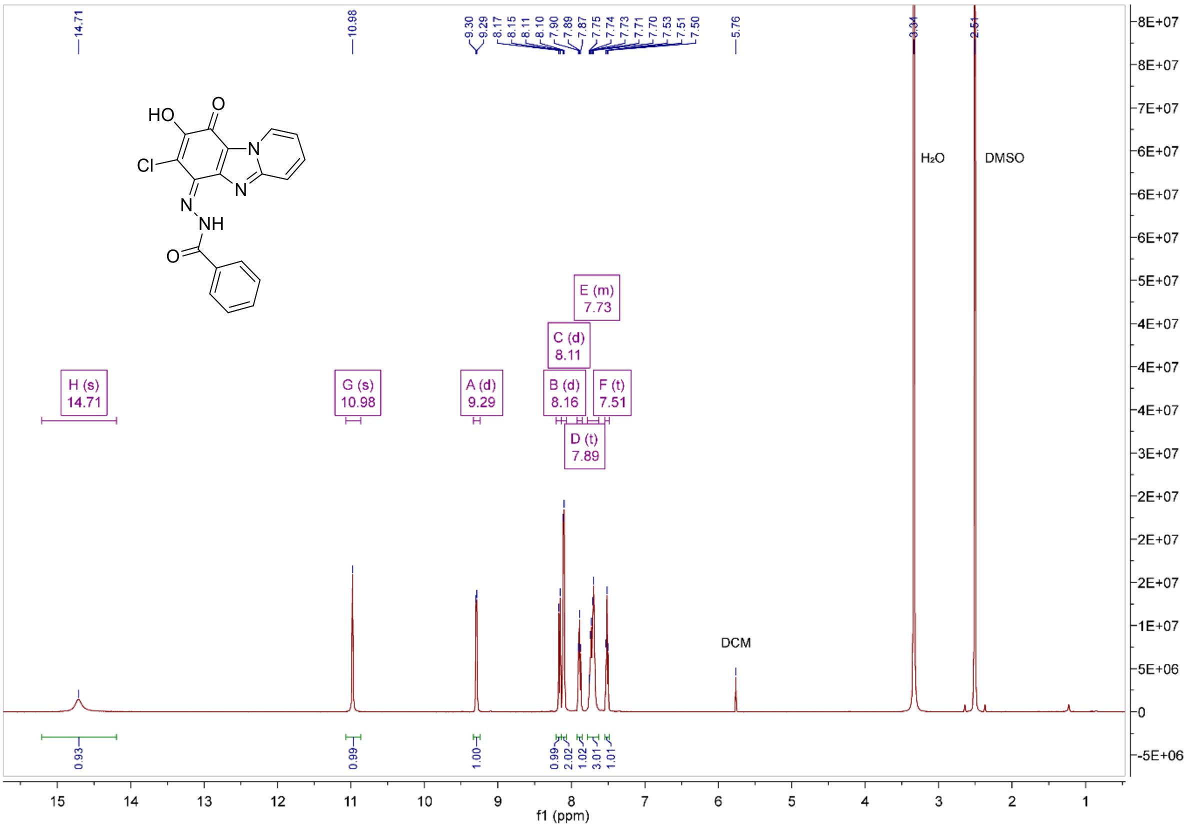

Figure S1. ^{1}H NMR (500 MHz, DMSO-d_6) spectrum of compound **3a**.

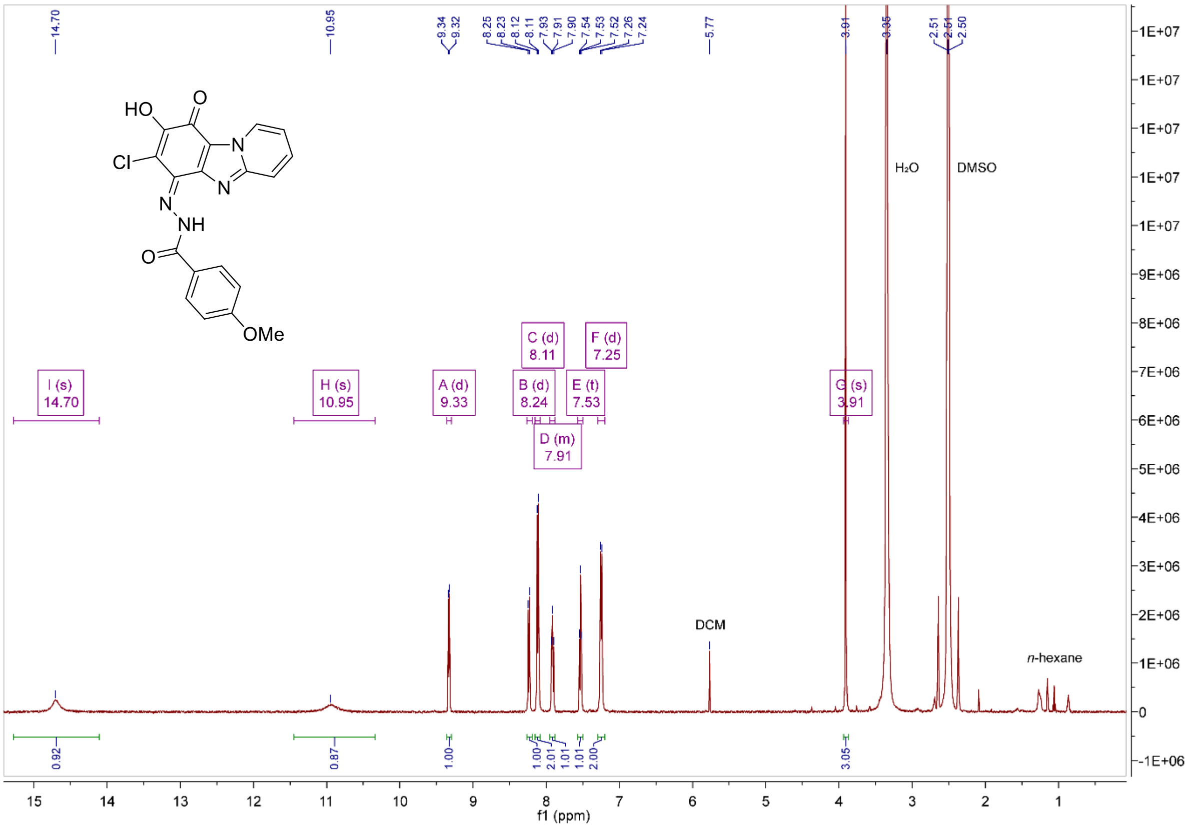

Figure S2. ^{1}H NMR (500 MHz, DMSO-d_6) spectrum of compound **3b**.

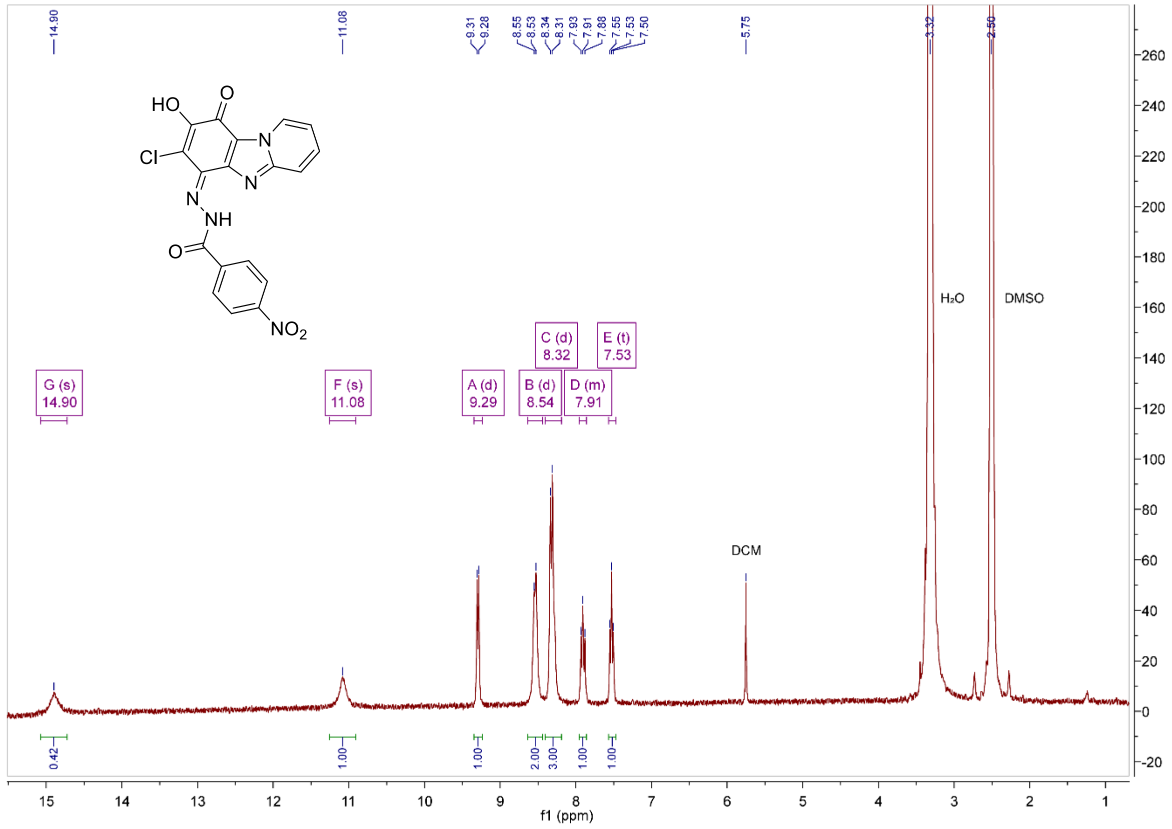

Figure S3. ^{1}H NMR (300 MHz, DMSO-d_6) spectrum of compound **3c**.

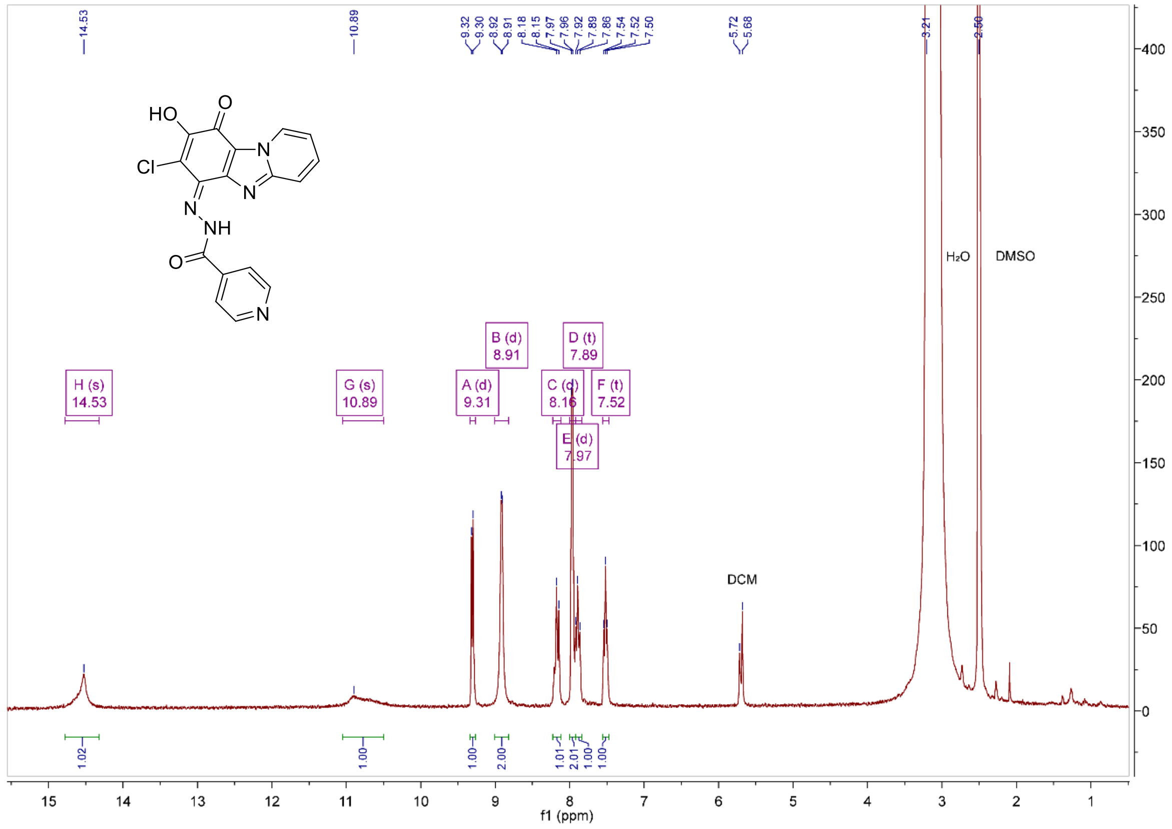

Figure S4. ^{1}H NMR (300 MHz, DMSO-d_6) spectrum of compound **3d**.

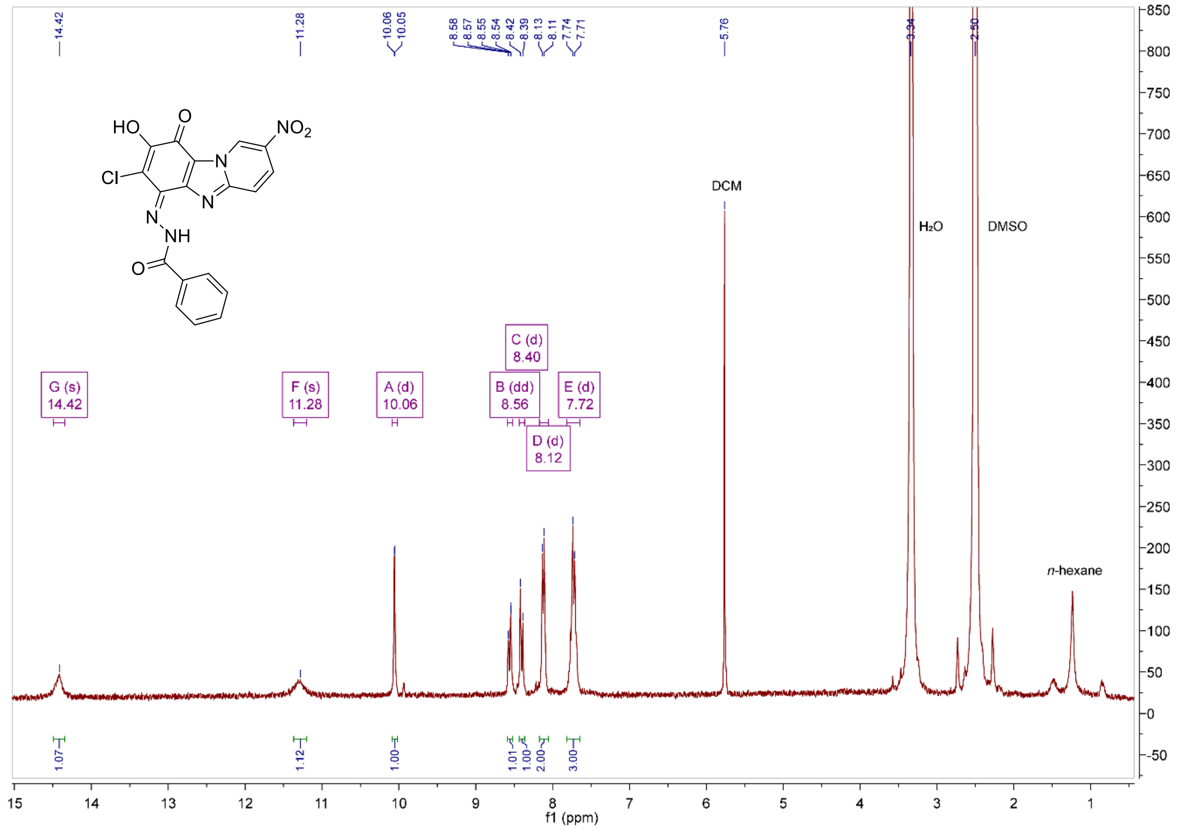

Figure S5. ^{1}H NMR (300 MHz, DMSO-d_6) spectrum of compound **3e**.

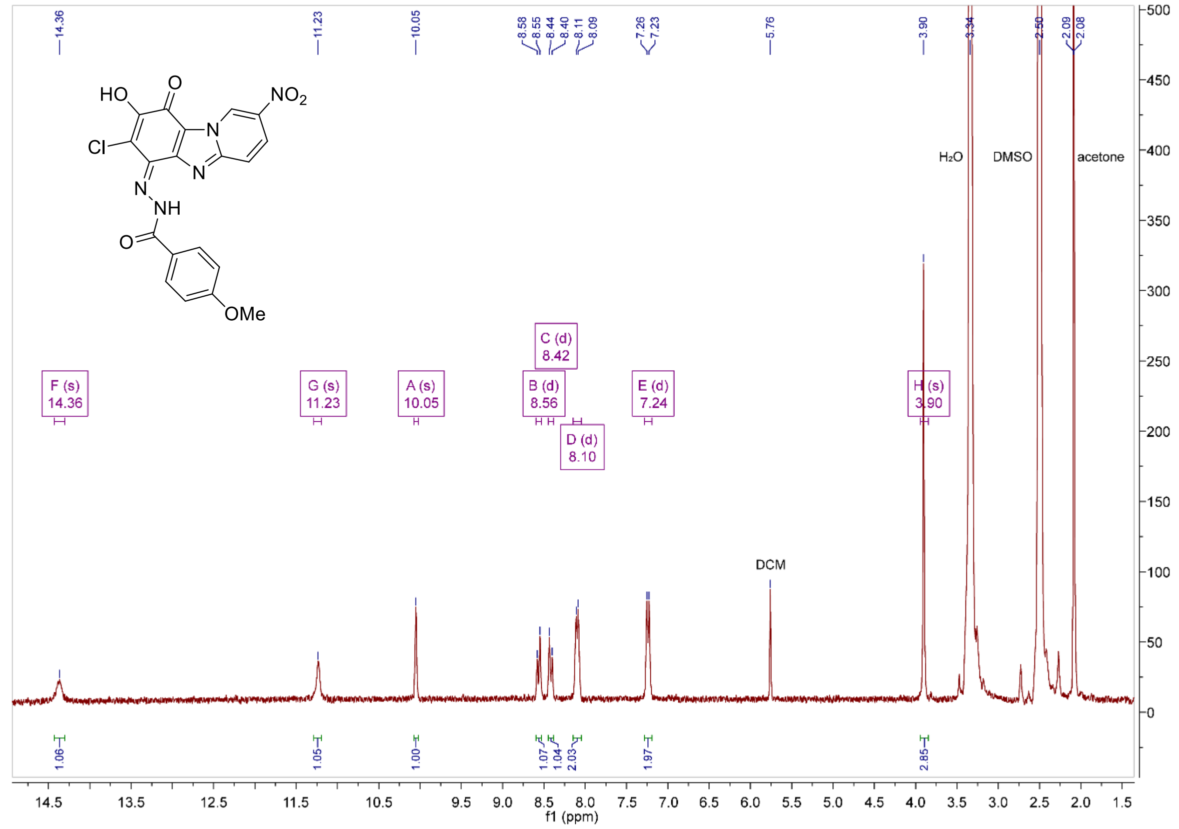

Figure S6. ^{1}H NMR (300 MHz, DMSO-d_6) spectrum of compound **3f**.

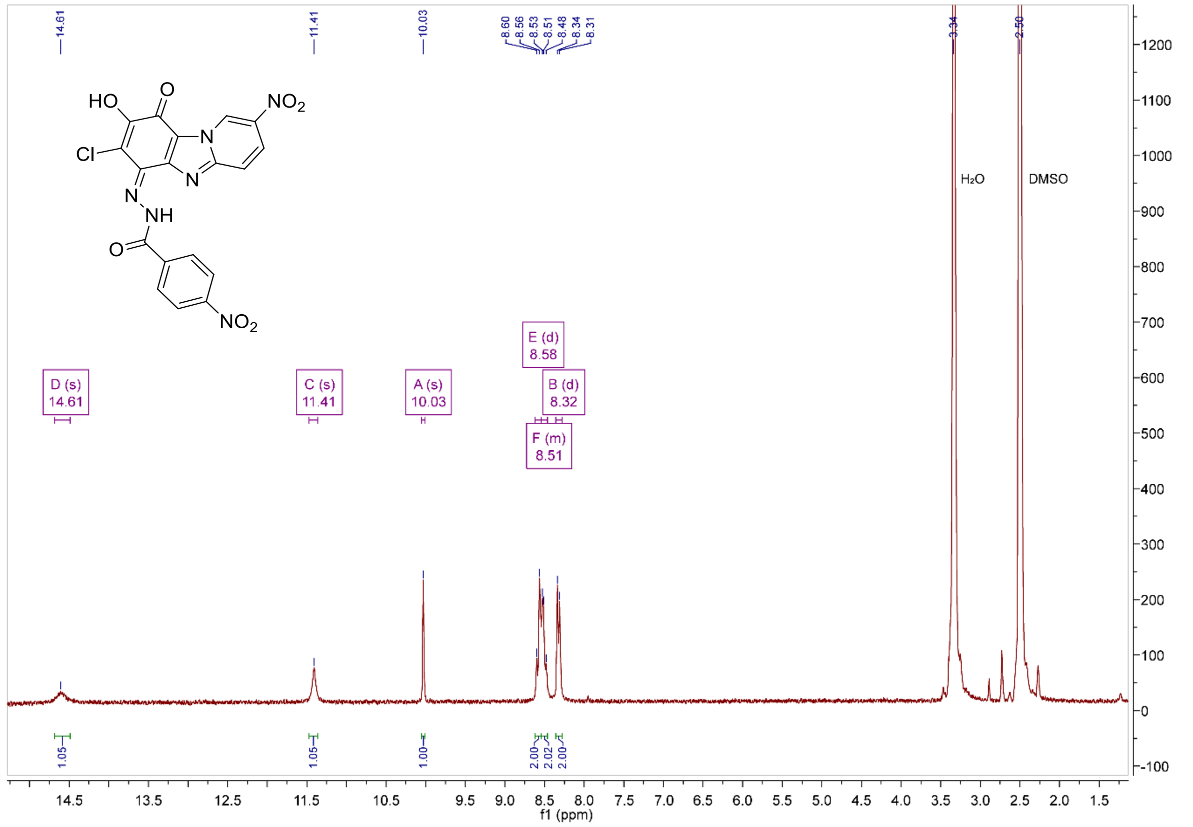

Figure S7. ^{1}H NMR (300 MHz, DMSO-d_6) spectrum of compound **3g**.

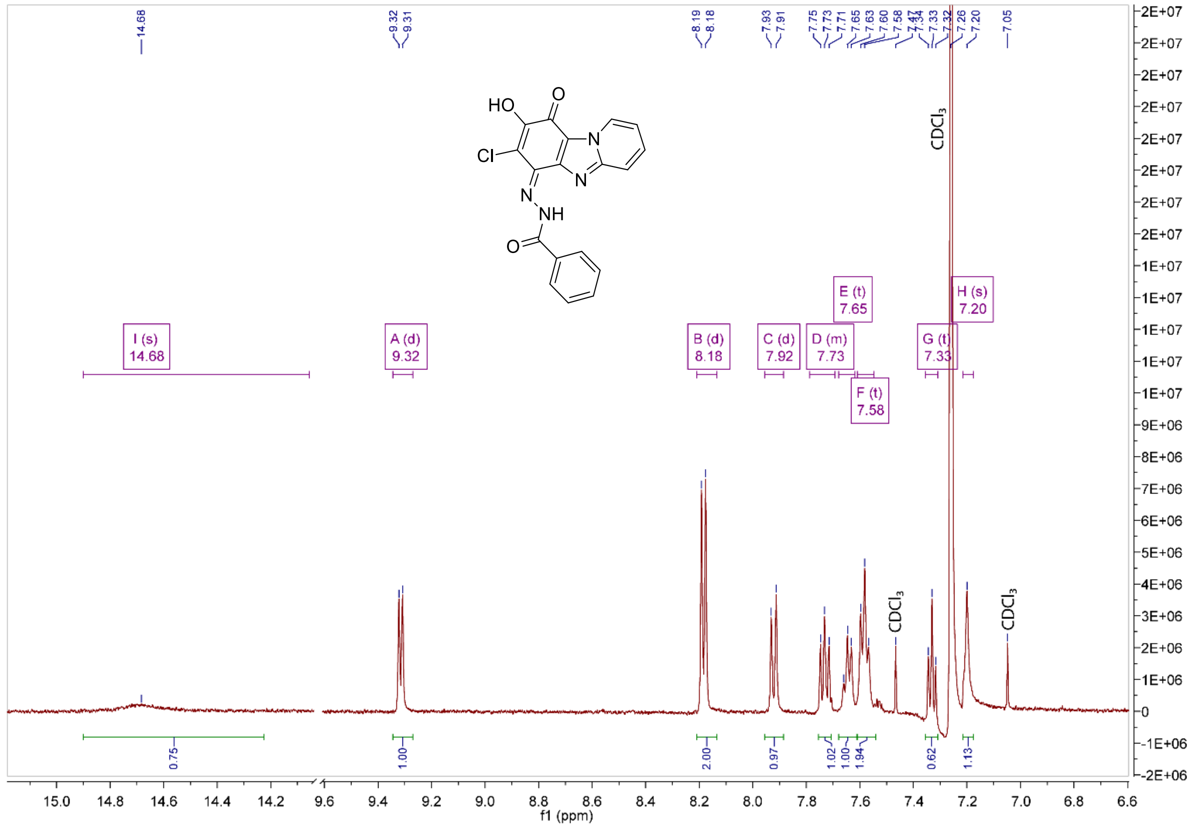

Figure S8. A fragment of ^{1}H NMR (500 MHz, $CDCl_3$) spectrum of compound **3a.**

Table S1. Substituents R_1 and R_2, the Hammett constant (for R_2) and chemical shifts of NH and OH protons in ^{1}H NMR spectra (DMSO-d_6) of the compounds **3a-c** and **3e-g**.

Compound	R_1	R_2	The Hammett constant of R_2 substituent (σ_p)	Chemical shift of NH proton (δ_{NH}), ppm	Chemical shift of OH proton (δ_{OH}), ppm
**3a**	H	H	0	14.71	10.98
**3b**	H	OMe	-0.27	14.70	10.95
**3c**	H	NO_2	0.78	14.90	11.08
**3e**	NO_2	H	0	14.42	11.28
**3f**	NO_2	OMe	-0.27	14.36	11.23
**3g**	NO_2	NO_2	0.78	14.61	11.41

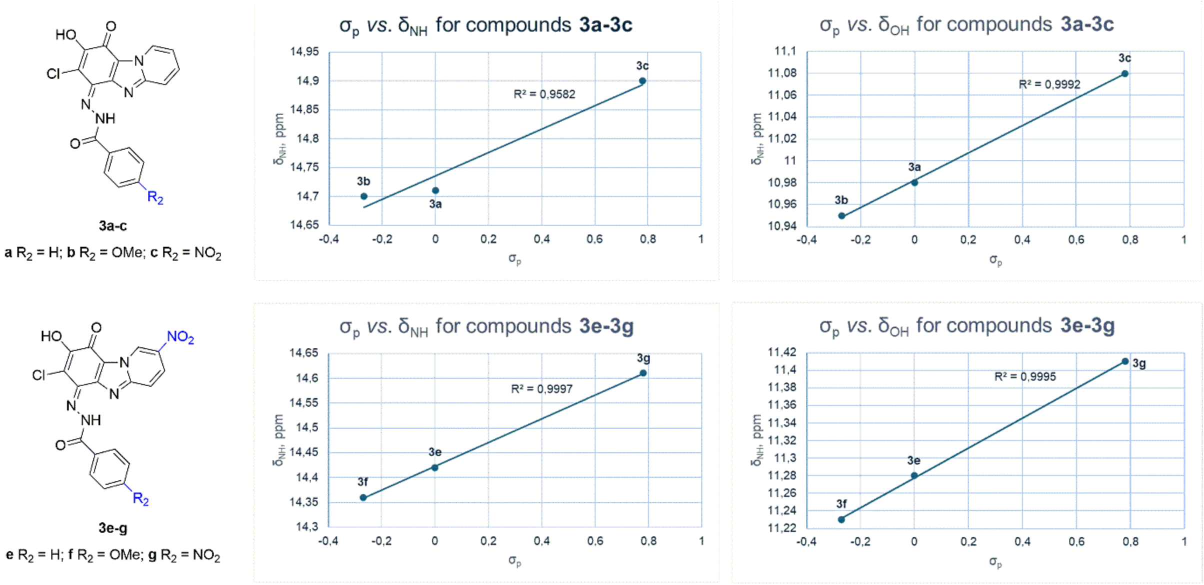

Figure S9. Structures of compounds **3a-c** and **3e-g** and correlations between the Hammett constants (σ_p) of substituent R_2 and the chemical shifts of NH proton (δ_{NH}) or OH proton (δ_{OH}) in ^{1}H NMR spectra (DMSO-d_6) of compounds **3a-c** and **3e-g**.

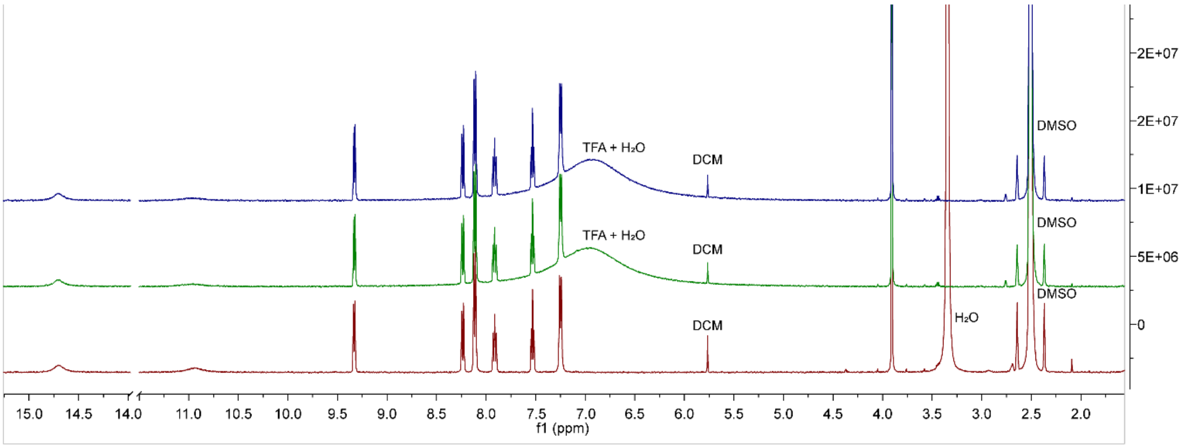

Figure S10. ^{1}H NMR (500 MHz, DMSO-d_6) spectra of compound **3b** (red line) after addition of TFA (green line) and UV irradiation (blue line).

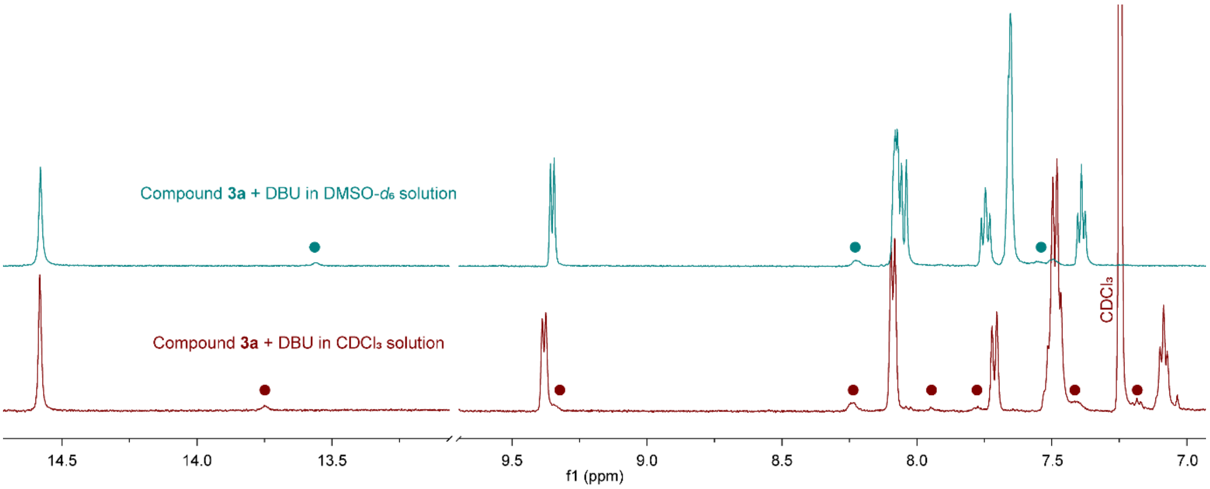

Figure S11. An expansion of ^{1}H NMR spectra (500 MHz) upon the addition of excess DBU to the solution of compound **3a** in DMSO-d_6 solution (blue line) or in $CDCl_3$ solution (red line). The signals of minor form of deprotonated compound **3a** are marked with circles.

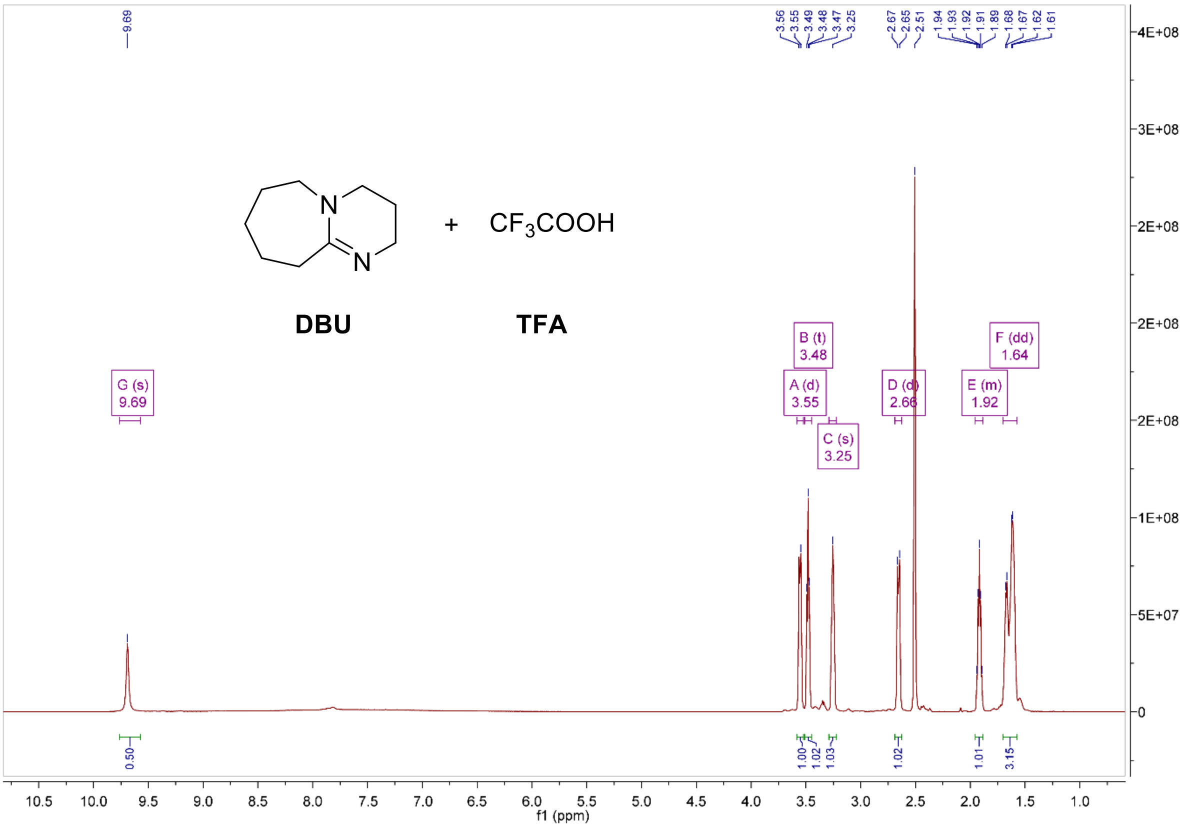

Figure S12. ^{1}H NMR (500 MHz, DMSO-d_6) spectrum of 1,8-diazabicyclo(5.4.0)undec-7-ene (**DBU**) and trifluoracetic acid (**TFA**) mixture.

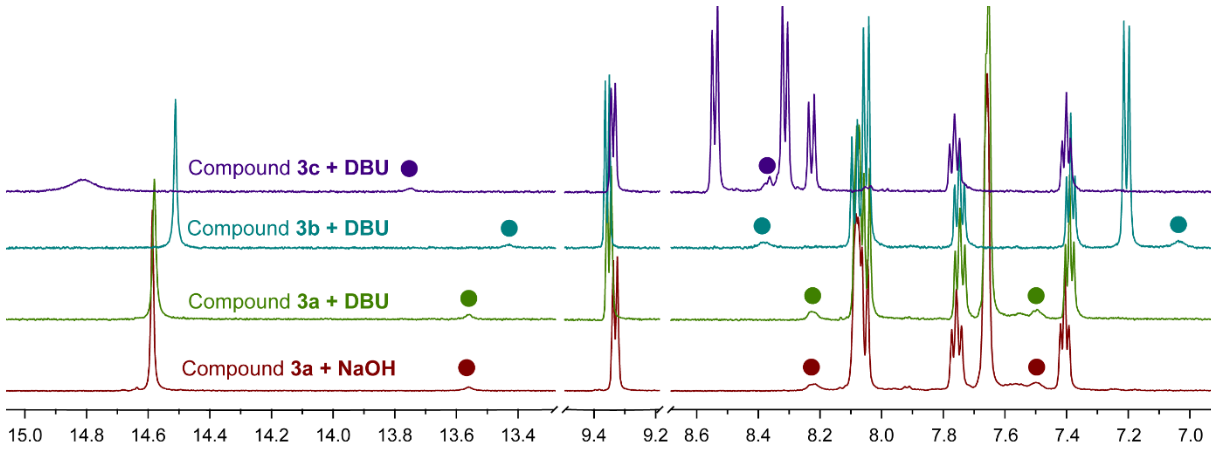

Figure S13. An expansion of ^{1}H NMR spectra (500 MHz, DMSO-d_6) upon the addition of excess NaOH (to the solution of compound **3a** (red line)) or excess of DBU (to the solutions of compound **3a** (green line), **3b** (blue line) or **3c** (purple line)). The signals of a minor form of deprotonated compounds **3a-c** are marked with circles.

2. Crystal Explorer: Hirshfeld Surfaces and Energy frameworks

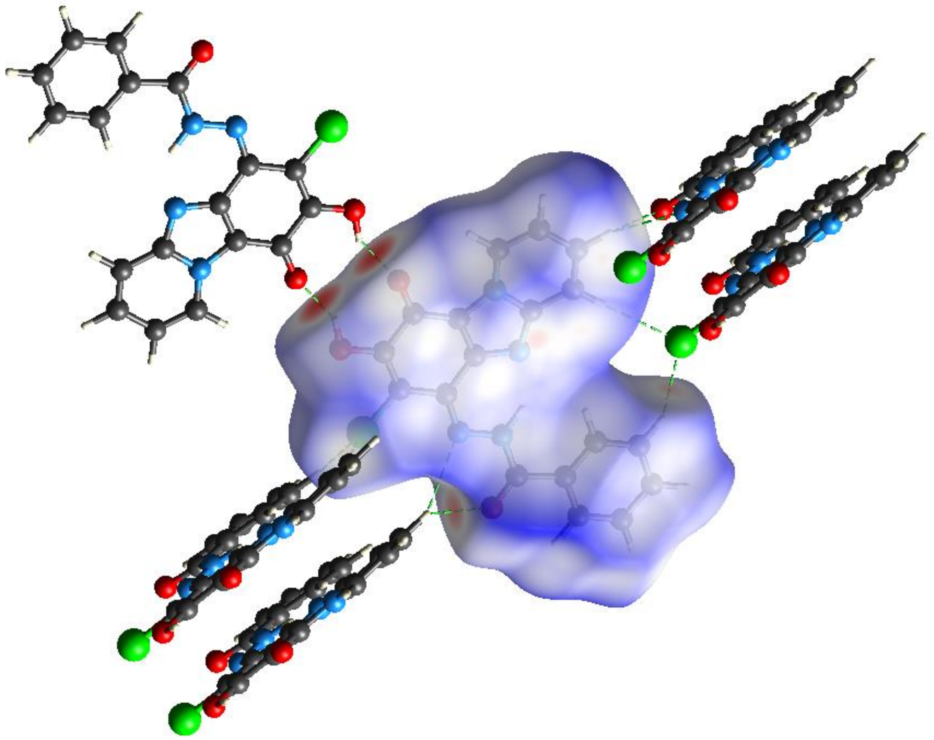

Figure S14. Hirshfeld surface for compound **3a.**

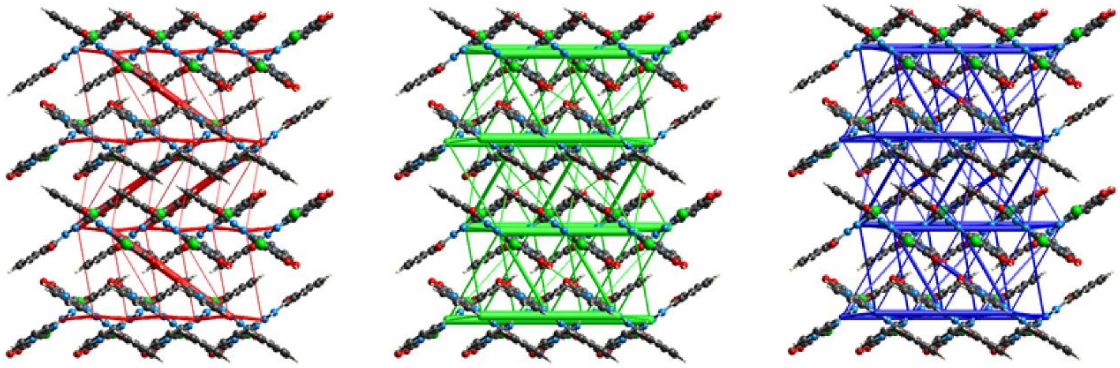

Figure S15. Energy frameworks of compound **3a**. Energy frameworks for separate electrostatic (red) and dispersion (green) contributions to the total nearest neighbor pairwise interaction energies (blue). All energies with a magnitude less than 5 kJ mol-1 have been omitted from the frameworks.

3. UV-Vis spectroscopy data

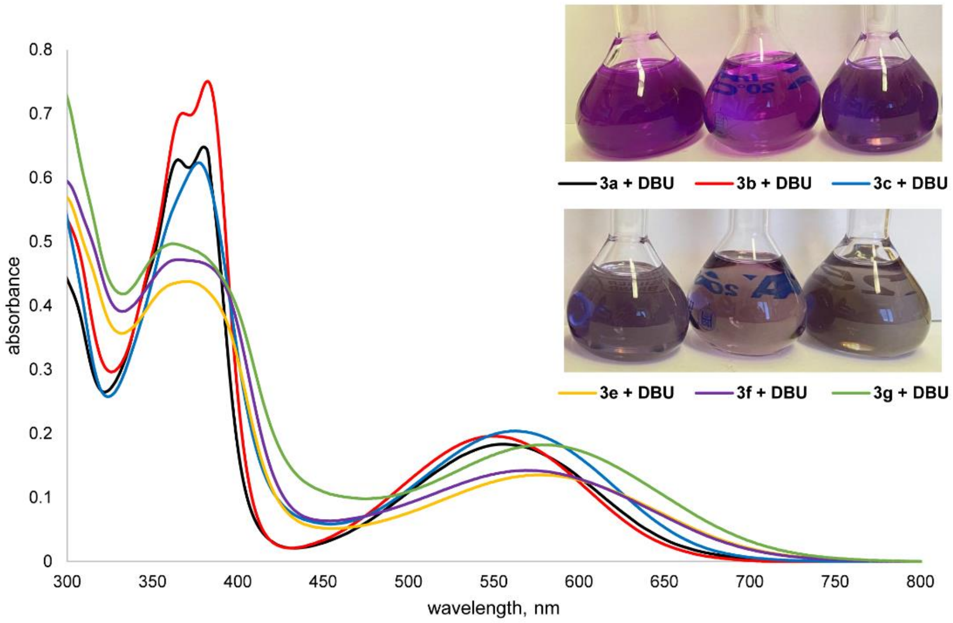

Figure S16. UV-Vis spectra of compounds **3a-c** and **3e-g** in $CHCl_3$ solution upon addition of DBU (excess).

Table S2. UV-Vis spectroscopy data of compounds **3a-c** and **3e-g** in the presence of DBU ($CHCl_3$).

Compound	λ_{max} (lg ε)
**3a**	365 (4.40), 380 (4.41), **556** (3.87)
**3b**	368 (4.45), 382 (4.48), **551** (3.89)
**3c**	377 (4.40), **563** (3.91)
**3e**	371 (4.24), **575** (3.73)
**3f**	365 (4.28), **569** (3.76)
**3g**	361 (4.30), **578** (3.86)

4. Redox properties

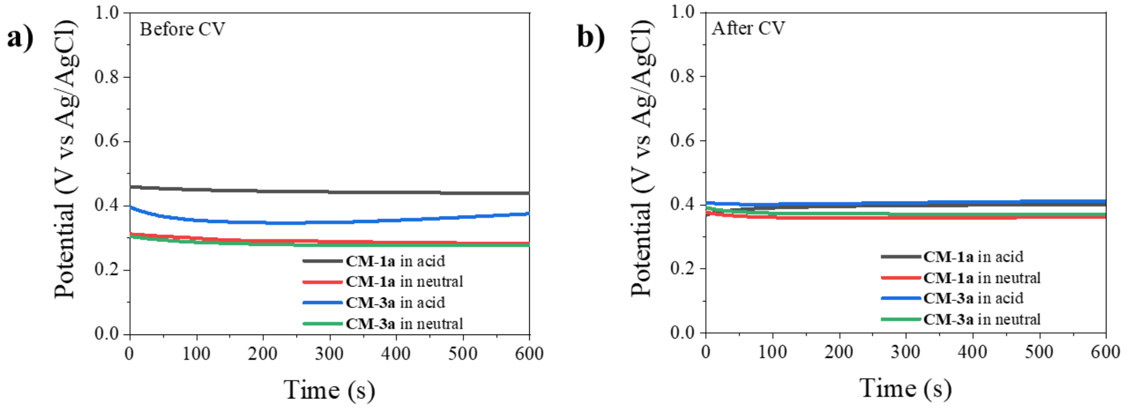

Figure S17. OCP measurements of samples **CM-1a** and **CM-3a** before and after CV measurements.

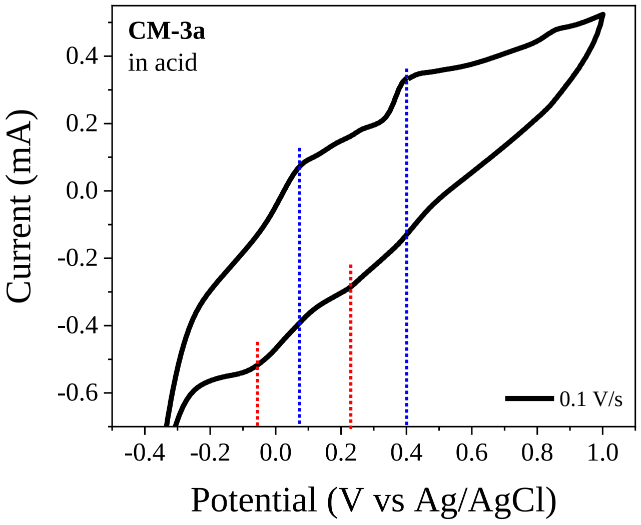

Figure S18. CV curve of sample **CM-3a** in acidic electrolyte at 0.1V/s scan speed.

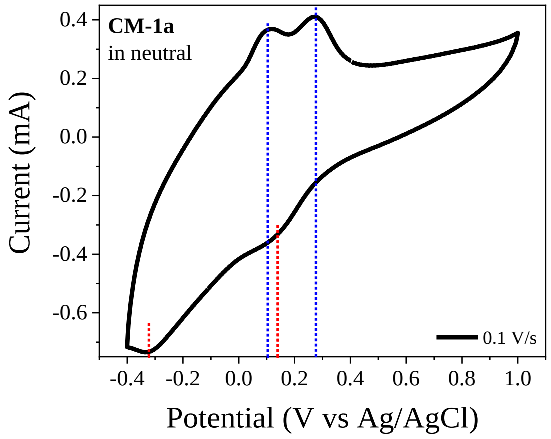

Figure S19. CV curve of sample **CM-1a** in neutral electrolyte at 0.1V/s scan speed.

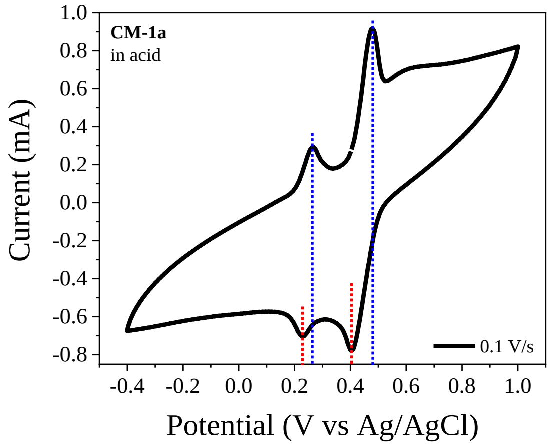

Figure S20. CV curve of sample **CM-1a** in acidic electrolyte at 0.1V/s scan speed.

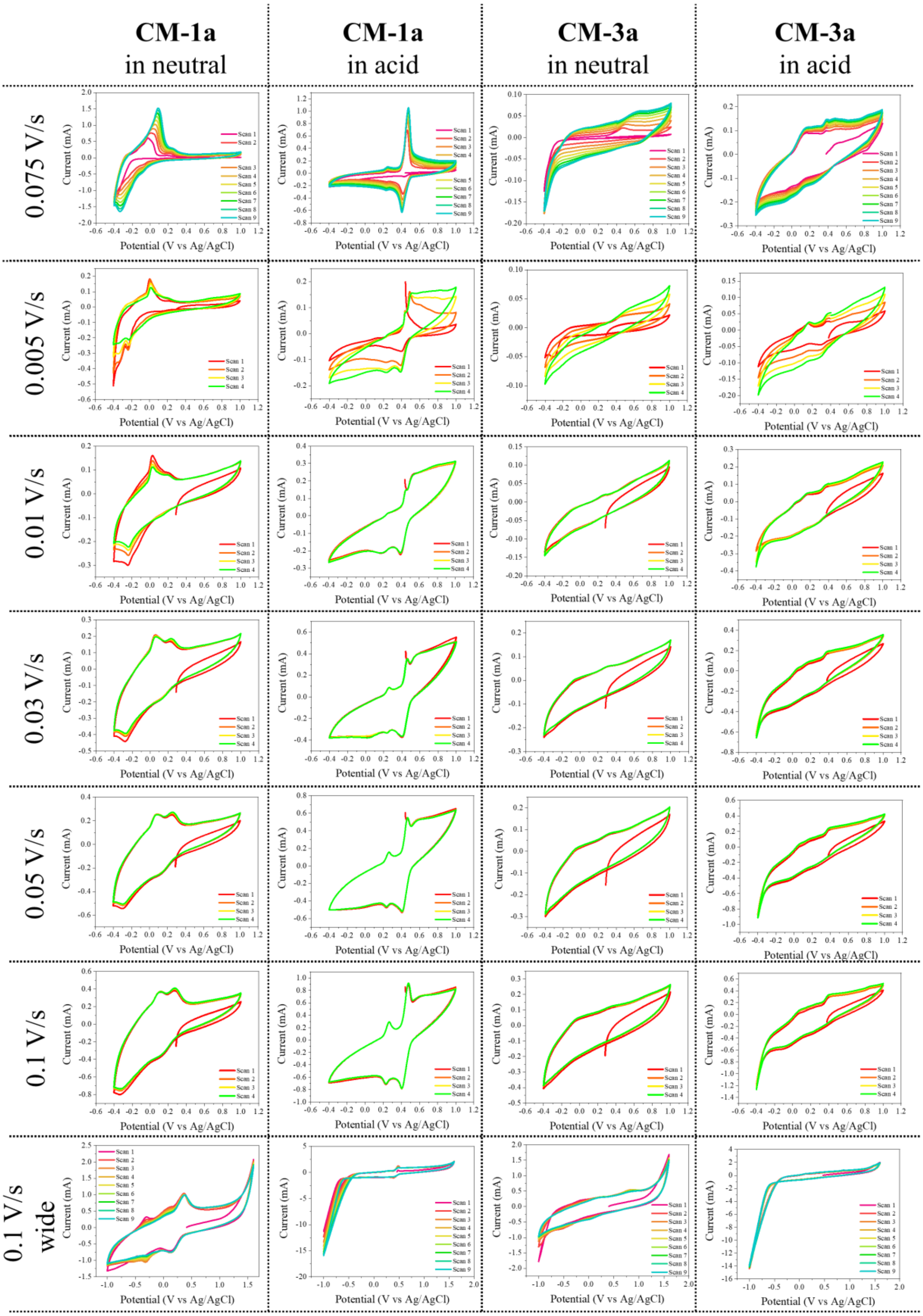

Figure S21. CV curves of samples **CM-1a** and **CM-3a** in neutral and acidic electrolyte at various scan speeds.

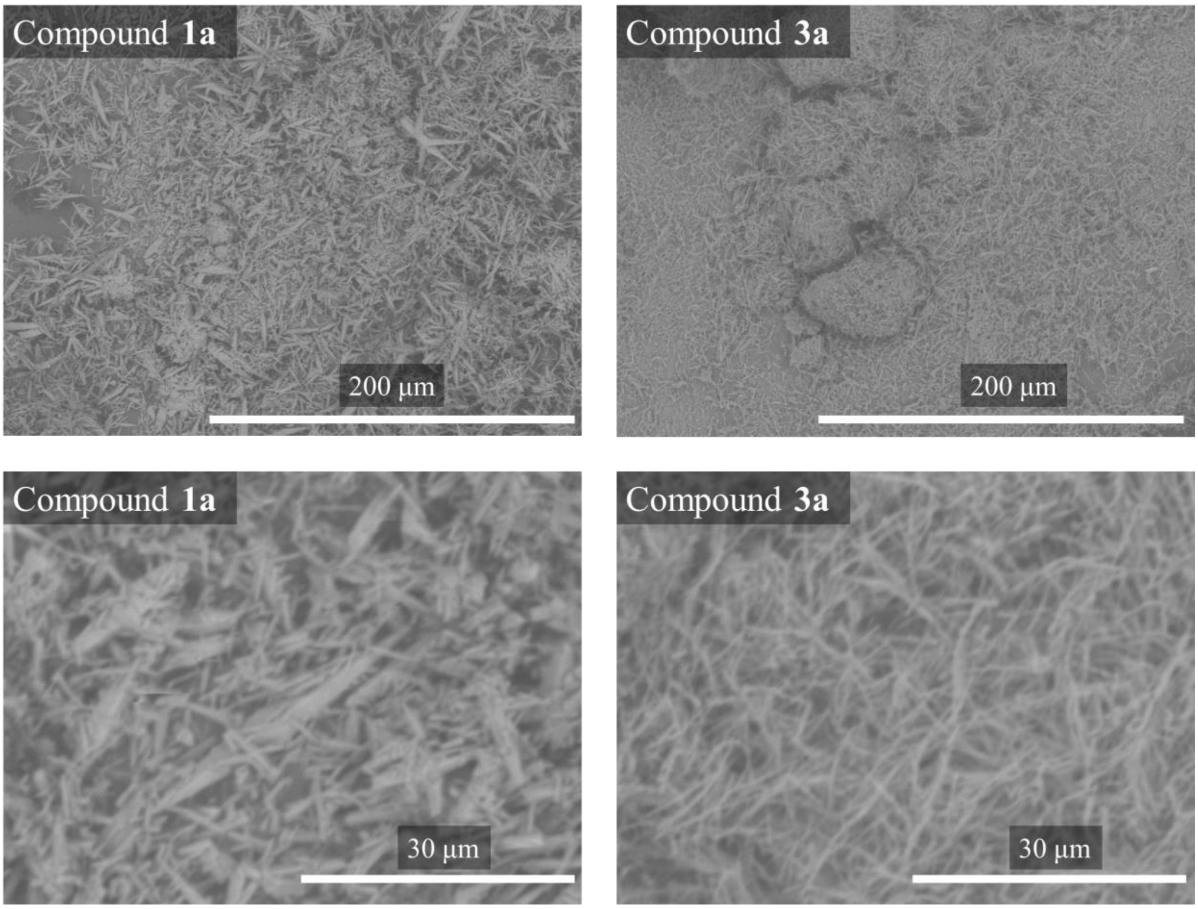

Figure S22. Scanning electron microscopy images of compounds **1a** and **3a** at different magnifications (x500 and x2500).
